# Alkaloids Mediate Multi-Level Modulation of Gastric Carcinogenesis: From Antibacterial and Anti-Inflammatory Actions to Antitumor Effects

**DOI:** 10.3390/ijms27177579

**Published:** 2026-08-24

**Authors:** Yanting Liu, Zijin Sun, Wanli Ouyang, Kunjing Liu, Chongyang Ma, Fang Lu, Qingguo Wang, Xueqian Wang, Fafeng Cheng

**Affiliations:** 1 School of Traditional Chinese Medicine, Beijing University of Chinese Medicine, Beijing 102488, China; 20240931018@bucm.edu.cn (Y.L.); 20210931013@bucm.edu.cn (Z.S.);; 2 School of Traditional Chinese Medicine, Capital Medical University, Beijing 100069, China; 3 School of Life Sciences, Beijing University of Chinese Medicine, Beijing 102488, China

**Keywords:** alkaloids, *Helicobacter pylori*, inflammation, precancerous lesions, gastric cancer

## Abstract

Gastric cancer is one of the malignancies with the highest incidence and mortality worldwide. *Helicobacter pylori* (*H. pylori*) infection is the primary driving factor in its development. The progression of gastric mucosal malignancy follows the Correa cascade model: “chronic non-atrophic gastritis → chronic atrophic gastritis (CAG) → intestinal metaplasia (IM) → dysplasia (Dys) → gastric cancer.” Currently, clinical management faces major challenges, including increasing antibiotic resistance in *H. pylori*, limited pharmacological options for gastric precancerous lesions, and treatment resistance and toxicity in established gastric cancer. This review synthesizes current evidence on BBR, COP, EPI, PAL, and JAT and organizes their reported actions into a three-tier intervention framework. At the first tier, etiologic and inflammatory interception, individual alkaloids suppress *H. pylori* persistence through direct antibacterial injury, urease inhibition, and modulation of bacterial virulence and antibiotic susceptibility, while attenuating infection-driven inflammatory and immune responses. At the second tier, modulation of precancerous mucosal progression, preclinical studies indicate that these compounds can ameliorate gastric glandular injury and may attenuate biological processes associated with progression toward intestinal metaplasia and dysplasia. At the third tier, antitumor and adjunctive intervention in established gastric cancer, alkaloids inhibit proliferation, induce cell-cycle arrest and apoptosis, suppress invasion and metastasis, and regulate non-coding RNA and epigenetic networks; BBR-centered preclinical studies further suggest potential chemosensitizing and supportive effects. This review integrates the five alkaloids BBR, COP, EPI, PAL, and JAT and systematically elucidates their mechanisms of action across the pathological continuum from *H. pylori* infection and chronic inflammation to precancerous lesions and ultimately gastric cancer. It establishes a stage-oriented, compound-specific analytical framework to clarify the pharmacological positioning of these compounds, identify priorities requiring further validation, and guide future mechanistic and translational research.

## 1. Introduction

Gastric cancer is a malignant tumor originating from the gastric mucosal epithelium. Based on tumor location, it can be classified into two major types: cardia gastric cancer and non-cardia gastric cancer. It is the fifth most commonly diagnosed cancer and the fifth leading cause of cancer-related death worldwide, with approximately 969,000 new cases and 660,000 deaths annually [1,2]. Gastric cancer is more common in Asia and in countries with a high Human Development Index (HDI) [3]. Due to its insidious early symptoms and the lack of specific screening biomarkers, approximately 70% of patients are diagnosed at an advanced metastatic stage, with a 5-year overall survival rate of less than 20% [4], resulting in a substantial global public health burden.

The development of gastric cancer is a complex process involving interactions among genetic susceptibility, environmental factors, microbial infection, and host immune responses. Although the number of new cases has increased with population growth, both the incidence rate and overall disease burden have shown a declining trend [5]. A large body of epidemiological, clinical, and experimental evidence has confirmed that *Helicobacter pylori* (*H. pylori*) infection is the primary initiating and driving factor of the Correa cascade, ultimately leading to gastric carcinogenesis [6]. In 1994, the World Health Organization’s International Agency for Research on Cancer (IARC) officially classified *H. pylori* as a Group I human carcinogen, establishing its causal relationship with gastric cancer [7]. More than half of the global population harbors *H. pylori* colonization in the gastric mucosa. In high-incidence regions, infection rates may exceed 70%, and globally, 76% of gastric cancer cases are attributable to *H. pylori* infection [8].

**Figure 1:**
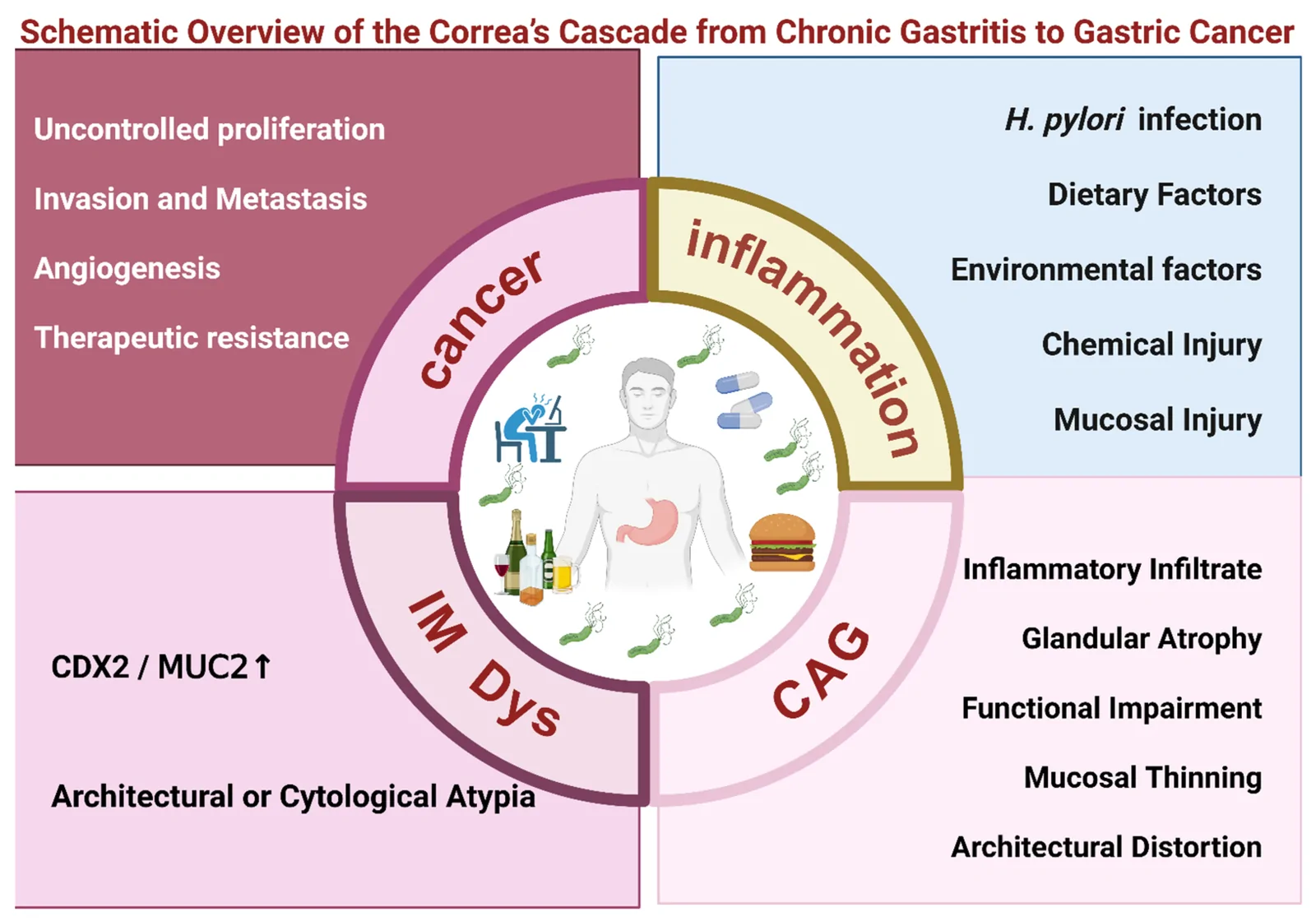
Schematic overview of the Correa cascade from chronic gastritis to gastric cancer. This diagram depicts the stepwise Correa cascade from *H. pylori* infection and chronic inflammatory infiltration to chronic atrophic gastritis (CAG), intestinal metaplasia (IM), dysplasia (Dys) and invasive gastric cancer, and summarizes the key histological alterations and molecular marker changes at each pathological stage.

Gastric carcinogenesis follows the classical Correa cascade, progressing from chronic non-atrophic gastritis to chronic atrophic gastritis (CAG), intestinal metaplasia (IM), dysplasia (Dys), and ultimately gastric cancer [9]. Among these stages, CAG, IM, and Dys are considered precancerous lesions of the stomach. This gradual pathological progression provides a critical window for early intervention in gastric cancer. Notably, the CAG and IM stages are regarded as key points for interrupting inflammation-to-cancer transformation and reducing gastric cancer incidence (Figure 1).

*H. pylori* colonizes the gastric mucosal layer and drives the Correa cascade through persistent infection. *H. pylori* virulence factors can impair mitochondrial function and increase intracellular ROS production. Elevated ROS can induce oxidative DNA lesions, including 8-OHdG, as well as single- and double-strand DNA breaks, thereby contributing to genomic instability. In parallel, virulence-associated signaling can activate oncogenic pathways and suppress tumor-suppressive programs [10,11]. *H. pylori* promotes malignant transformation of gastric epithelial cells through multiple complex molecular mechanisms [12]. For instance, *H. pylori* infection upregulates TGF-β1 and phosphorylated Smad2 (p-Smad2), thereby activating the epithelial–mesenchymal transition (EMT) pathway [13].

Virulence factors constitute the core basis of *H. pylori*-mediated carcinogenesis, among which cytotoxin-associated gene A (CagA) [14] and vacuolating cytotoxin A (VacA) [15] are the most critical, exerting potent disruptive effects on host cells [16]. CagA activates the TERT/Wnt/β-catenin signaling pathway, promoting chief cell dedifferentiation and the development of spasmolytic polypeptide-expressing metaplasia (SPEM) in the gastric mucosa [17]. In addition, CagA suppresses T-cell activity, thereby facilitating immune evasion in gastric cancer [18]. Crucially, by translocating CagA into gastric epithelial cells through the type IV secretion system, *H. pylori* endows normal cells with a range of phenotypic cancer hallmarks, including sustained proliferation, evasion of growth suppressors, invasiveness, resistance to cell death, and genomic instability, thereby significantly driving gastric tumorigenesis [19]. VacA induces host cell vacuolation, apoptosis, and immune dysregulation through the formation of hexameric pores, thereby promoting gastric mucosal injury and the persistence of chronic inflammation. For example, the VacA/TNFAIP3/TRAF1 signaling axis promotes pyroptosis in *H. pylori*-infected tissues, leading to atrophic morphological changes in gastric epithelial cells and subsequently driving sustained malignant transformation [20].

Second, *H. pylori*-induced chronic inflammation is a key driver of inflammation-to-cancer transformation. Persistent *H. pylori* infection activates core pro-inflammatory signaling pathways such as NF-κB and MAPK [21], resulting in increased production of pro-inflammatory cytokines, including IL-1β, IL-6, and TNF-α [22], and contributing to chronic mucosal injury, glandular atrophy, and metaplastic remodeling. In addition, *H. pylori* promotes tumor progression by inducing EMT and acquisition of cancer stem cell-like properties, thereby enhancing tumor cell invasiveness and metastatic potential [23]. *H. pylori* infection mediates EMT processes in the gastric epithelium; for example, CagA-induced EMT-like changes are associated with enhanced cancer stem cell-like traits, increased cellular invasiveness and migratory capacity, and ultimately contribute to tumor development [24,25].

Although the World Health Organization has classified *H. pylori* as a Group I carcinogen [7], clinical eradication of *H. pylori* still faces major challenges, including rising antibiotic resistance and a lack of targeted therapeutic strategies [26]. Antibiotic resistance among *H. pylori* strains has increased globally, compromising the efficacy of several commonly used eradication regimens [27,28]. Even with bismuth-containing quadruple therapy, eradication rates of multidrug-resistant *H. pylori* strains remain relatively low, and treatment is often accompanied by gut microbiota dysbiosis and gastrointestinal adverse effects. At present, effective pharmacological options remain limited, representing a major bottleneck in secondary prevention of gastric cancer.

Compared with single-target chemotherapeutic agents, natural isoquinoline alkaloids have attracted increasing pharmacological interest because of their diverse and partially overlapping molecular targets and signaling pathways. However, the strength of evidence varies substantially among individual compounds, and evidence regarding synergistic effects, resistance-modifying properties, and long-term safety remains incomplete. These properties endow them with remarkable research and translational value in the prevention and treatment of chronic inflammatory diseases and malignancies [29]. Accumulating evidence has documented their pronounced efficacy in anti-infection [30], anti-inflammation [31], immunomodulatory, and neuroregulatory effects [32], as well as their well-recognized therapeutic potential across multiple pathological stages of gastric carcinogenesis, including gastric precancerous lesions and established gastric cancer.

Protoberberine-type isoquinoline alkaloids represent the structural subclass of isoquinoline alkaloids with the most diverse pharmacological activities [33,34]. Among them, berberine (BBR), coptisine (COP), epiberberine (EPI), palmatine (PAL), and jatrorrhizine (JAT) are the five most representative bioactive monomers of this subclass, each exerting a broad spectrum of pharmacological effects [35,36]. As a naturally occurring quaternary ammonium alkaloid, BBR is one of the most extensively investigated compounds among the five and has demonstrated broad antibacterial activity [37,38]. BBR has demonstrated significant therapeutic effects in anti-inflammatory [39], cardiovascular protection [40], and neuroprotection [41]. Moreover, its mechanisms of action across multiple stages of gastric mucosal inflammation-to-cancer transformation have been extensively investigated [42].

COP shares the protoberberine core scaffold with BBR [43], and exhibits diverse pharmacological activities, including antitumor, anti-inflammatory, metabolic-regulatory, and antioxidant effects [44]. Preclinical studies have reported antibacterial activity of EPI [45] through modulation of the *H. pylori* urease system [46,47] together with effects in experimental CAG and gastric cancer models [48,49,50]. PAL is another natural isoquinoline alkaloid verified to exert multiple pharmacological activities [51,52], including antioxidant [53], anti-inflammatory [54], and gut microbiota-regulating effects [55]. JAT is a protoberberine-type isoquinoline alkaloid. Although systematic investigations into this monomer remain relatively limited to date, accumulating reports have demonstrated its neuroprotective, anti-tumor, antibacterial, anti-rheumatoid arthritis, hypoglycemic, lipid-lowering, and anti-obesity activities [56] (Table 1).

Among these five alkaloids, BBR is by far the most extensively investigated compound, whereas the evidence for COP, EPI, PAL, and particularly JAT is considerably more limited and stage specific. Accordingly, the five compounds should not be regarded as pharmacologically or evidentially equivalent. Against this background, the present review compares their compound-specific evidence profiles across different stages of gastric carcinogenesis, including *H. pylori* infection, chronic inflammation, gastric precancerous lesions, and established gastric cancer. We further conduct an in-depth analysis of the interventional effects and molecular mechanisms of these compounds across distinct pathological stages of gastric carcinogenesis, with particular attention to their compound-specific and stage-specific evidence profiles. This review aims to provide a stage-oriented framework for evaluating the pharmacological evidence on these five alkaloids and to identify priorities for future translational investigation.

Literature search approach: Relevant literature was identified primarily through PubMed, Web of Science, and Scopus, covering the period from database inception through 31 July 2026. The core search strategy combined the five alkaloids discussed in this review with terms representing the pathological continuum of gastric carcinogenesis: (berberine OR coptisine OR epiberberine OR palmatine OR jatrorrhizine) AND (“*Helicobacter pylori*” OR gastritis OR “chronic atrophic gastritis” OR “gastric atrophy” OR “intestinal metaplasia” OR dysplasia OR “gastric precancerous lesions” OR “gastric cancer” OR “gastric carcinoma” OR “stomach neoplasms”). Experimental, mechanistic, and available clinical studies were considered when they directly addressed one or more of the pathological stages or pharmacological mechanisms covered in this review. Studies directly relevant to gastric disease progression or to the pharmacological actions of the five alkaloids were prioritized for mechanistic synthesis. Because this review was not prospectively designed or documented as a PRISMA-based systematic review, formal screening counts and a PRISMA flow diagram are not provided.

Quantitative pharmacological parameters were retained when they were directly interpretable within individual experimental contexts. However, MICs, enzyme-inhibition parameters, cell-viability measures, animal doses, and other pharmacodynamic endpoints were not pooled or ranked across studies because they were generated using heterogeneous bacterial strains, cell lines, assay systems, exposure durations, dosing regimens, and outcome definitions.

## 2. Anti-*H. pylori* Actions of Isoquinoline Alkaloids: Direct Bacterial Injury, Urease Inhibition, and Modulation of Antibiotic Susceptibility

### 2.1. Direct Antibacterial Effects on H. pylori Structural Integrity and Viability

Isoquinoline alkaloids exert direct antibacterial activity against *H. pylori* through multiple mechanisms, although the nature and strength of the available evidence differ substantially among individual compounds. BBR and PAL have both been reported to inhibit the growth of *H. pylori* [57,58]. Notably, recent medicinal-chemistry studies based on the BBR/protoberberine scaffold have generated derivatives with markedly enhanced anti-*H. pylori* activity. Some of these derivatives alter bacterial membrane permeability, interact with *H. pylori* DNA, and simultaneously inhibit urease activity [57]. Because these observations were obtained primarily with structurally optimized BBR derivatives rather than free BBR itself, they should be interpreted as evidence supporting the antibacterial potential of the protoberberine scaffold rather than as direct mechanistic evidence for native BBR.

More direct evidence of bacterial structural disruption has been obtained for COP. COP inhibits *H. pylori* viability in a concentration- and time-dependent manner and induces DNA fragmentation, phosphatidylserine externalization, and membrane damage, indicating loss of bacterial cellular integrity [59]. Transmission electron microscopy and complementary cellular assays further support prominent morphological and structural injury following COP exposure. In addition to its direct antibacterial action, COP decreases CagA expression and reduces CagA translocation into gastric epithelial cells, thereby attenuating virulence-associated host-cell injury [59]. EPI also exhibits direct bacteriostatic and bactericidal effects against *H. pylori*. MIC, MBC, bacterial growth-kinetic, resistance-development, and transmission electron microscopy assays demonstrated that EPI markedly inhibits bacterial growth and induces bacterial fragmentation, without detectable resistance development under the experimental conditions [46]. These structural alterations were accompanied by reduced bacterial colonization and gastric inflammatory injury in experimental models [46].

Collectively, the available evidence indicates that direct bacterial injury constitutes an important component of the anti-*H. pylori* activity of protoberberine alkaloids. However, direct morphological or structural evidence is currently strongest for COP and EPI, whereas comparable evidence for the remaining compounds is less extensive. Therefore, disruption of bacterial structural integrity should not be regarded as a uniformly established mechanism for all five alkaloids.

### 2.2. Inhibition of Urease Activity and Acid Adaptation

*H. pylori* urease (HPU) is a major determinant of bacterial survival in the acidic gastric niche. The enzyme hydrolyzes urea to generate ammonia and carbon dioxide, thereby creating a locally buffered microenvironment that facilitates bacterial survival and persistent colonization within the gastric mucus layer [60,61]. Formation of catalytically active urease additionally requires a coordinated maturation process involving accessory proteins such as UreG, UreF, and UreD, which participate in nickel delivery and incorporation into the urease complex [62,63]. Therefore, inhibition of either urease catalytic activity or accessory-protein-dependent maturation represents an important non-classical strategy for suppressing *H. pylori* persistence. Recent functional mapping of the *H. pylori* urease system further demonstrated that the catalytic pocket is highly evolutionarily conserved and that disruption of urease activity markedly compromises bacterial colonization and virulence. Importantly, urease inhibition can also potentiate antibiotic activity, supporting urease as a relatively resistance-refractory antibacterial target [64].

Several alkaloids discussed in this review interfere with the urease system at different regulatory levels. BBR and BBR-containing multicomponent preparations have been shown to inhibit *H. pylori* urease activity. Comparative enzymatic analyses implicated interactions with sulfhydryl groups of urease in this inhibitory effect [65]. These observations support urease inhibition as one component of the anti-*H. pylori* activity associated with BBR-containing preparations, although highly specific structural interpretations of BBR–urease binding require further validation. COP has a more extensively characterized dual action on the urease system. In addition to directly inhibiting *H. pylori* urease, COP interacts with the nickel-containing catalytic machinery and interferes with urease maturation by inhibiting the accessory protein UreG. COP suppresses UreG activity and dimer formation and promotes nickel dissociation from UreG dimers, thereby impairing the generation of catalytically competent urease [66]. EPI also interferes with urease through multiple complementary mechanisms. It directly inhibits *H. pylori* urease activity and downregulates expression of the urease structural subunit gene *ureB* [46,47]. EPI has been reported to exhibit strong urease-inhibitory activity in comparison with acetohydroxamic acid in experimental assays [47]. In addition, inhibition of HPU by EPI reduces HPU-induced apoptosis and inflammatory injury in gastric epithelial cells, providing a functional link between bacterial urease inhibition and protection against host-cell damage [46]. PAL also inhibits *H. pylori* growth, at least in part through suppression of bacterial urease [58]. Enzyme kinetic analyses demonstrated that PAL inhibits both *H. pylori* urease and jack-bean urease in a concentration-dependent, reversible, and noncompetitive manner. Thiol-protection and dithiothreitol (DTT)-reactivation experiments further implicated sulfhydryl groups in the active site in this inhibitory effect [58]. Molecular docking suggested interactions between PAL and residues associated with the urease mobile flap, providing an additional structural basis for inhibition [58]. Structural optimization of PAL has also generated derivatives with enhanced anti-*H. pylori* activity and urease-inhibitory properties [67].

Collectively, these findings demonstrate that protoberberine alkaloids can interfere with *H. pylori* acid adaptation at several distinct levels, including direct inhibition of urease catalytic activity, suppression of urease structural-gene expression, and disruption of accessory-protein-dependent urease maturation. These mechanisms provide a biological rationale for targeting urease as an antibiotic-independent vulnerability of *H. pylori*, although the specific molecular targets and inhibitory potency differ substantially among individual alkaloids.

### 2.3. Modulation of Antibiotic Susceptibility and Potentiation of H. pylori Eradication Therapy

Antibiotic resistance remains a major obstacle to successful *H. pylori* eradication. Resistance is mediated by multiple mechanisms, including alterations in antibiotic targets, reduced drug accumulation, and increased activity of bacterial efflux systems. Among the latter, overexpression of the *hefA* efflux-pump gene has been associated with antimicrobial resistance in *H. pylori* [68]. Therefore, modulation of bacterial drug susceptibility represents a potentially complementary role of alkaloids in addition to their direct antibacterial activity.

BBR may modulate antibiotic susceptibility partly through regulation of the *hefA* efflux system. In multidrug-resistant *H. pylori* isolates, exposure to BBR at sub-inhibitory concentrations reduced the MICs of amoxicillin and tetracycline in selected strains. In the majority of isolates showing decreased amoxicillin MICs, *hefA* mRNA expression was also reduced [69]. These findings suggest that BBR may partially restore susceptibility to selected antibiotics through mechanisms associated with downregulation of *hefA*, although this relationship was not uniform across all resistant strains [69]. COP also exhibits activity against clarithromycin-resistant *H. pylori* [70]. More recent mechanistic analyses indicate that COP can interfere with bacterial oxidative-stress adaptation in resistant strains. Transcriptomic and functional experiments identified *katA*, which encodes catalase, as an important regulatory node associated with COP activity. Downregulation of *katA* compromises bacterial survival under oxidative stress and is accompanied by reduced bacterial adhesion and attenuation of oxidative and inflammatory injury in gastric epithelial cells [70]. These findings suggest that the activity of COP against resistant *H. pylori* may involve bacterial fitness and stress-adaptation mechanisms distinct from classical antibiotic targets.

Beyond these mechanistic observations, BBR has been evaluated clinically as an adjunct to antibiotic-based *H. pylori* eradication therapy. A BBR-containing triple regimen achieved an eradication rate of 90.1% in one clinical study, compared with 86.4% for the corresponding bismuth-containing regimen [71]. In addition, BBR-containing amoxicillin-based quadruple therapy demonstrated non-inferiority to a tetracycline–furazolidone-containing regimen in rescue treatment [72]. Supplementation of conventional triple therapy with BBR has also been associated with improved eradication efficacy [73], and additional clinical studies have reported comparable eradication performance with favorable tolerability for BBR-containing regimens [74].

Taken together, these findings support a potential role for BBR as an adjunct to conventional *H. pylori* eradication therapy. However, improved eradication efficacy in combination regimens should not be interpreted as direct clinical evidence that BBR reverses a defined molecular mechanism of antibiotic resistance in vivo. Rather, the available data support a broader interpretation in which BBR may modulate bacterial susceptibility while providing additional antibacterial activity within multidrug eradication regimens.

Emerging formulation-based approaches may further extend the activity of alkaloids against drug-resistant and biofilm-associated *H. pylori*. For example, a recently developed mucoadhesive lignin–liposome nanocarrier containing COP exhibited an MIC of 32 μg/mL and an MBC of 64 μg/mL against *H. pylori*, eradicated more than 92% of planktonic bacteria, and disrupted approximately 89% of established biofilms within 24 h [75]. Importantly, these effects reflect the combined properties of the COP-loaded delivery system and should not be interpreted as evidence that free COP alone possesses equivalent antibiofilm activity.

**Figure 2:**
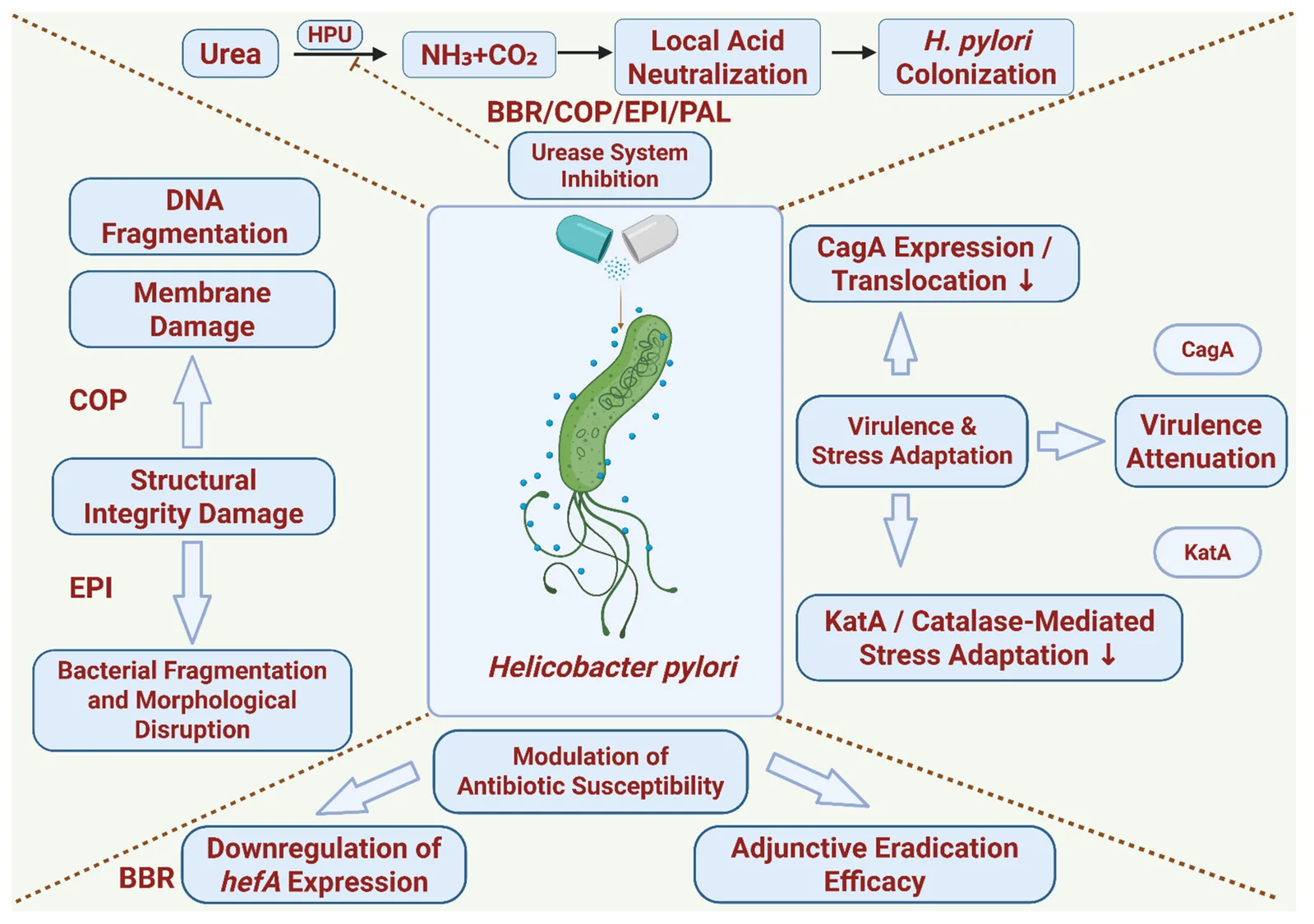
Multi-target anti-*H. pylori* actions of protoberberine alkaloids. Specific alkaloids act through distinct antibacterial mechanisms. COP and EPI induce direct bacterial structural injury, including membrane damage, DNA fragmentation, and bacterial fragmentation. BBR, COP, EPI, and PAL interfere with the urease system through inhibition of catalytic activity, regulation of *ureB* expression, or disruption of UreG-dependent urease maturation, thereby impairing acid adaptation and bacterial persistence. COP additionally attenuates CagA-associated pathogenicity and *KatA*/catalase-mediated oxidative-stress adaptation. BBR may modulate antibiotic susceptibility partly through downregulation of the *hefA* efflux system, while BBR-containing regimens have demonstrated adjunctive efficacy in clinical eradication studies. The strength and type of evidence differ among individual alkaloids, and the illustrated mechanisms should not be interpreted as uniformly established for all five compounds. Arrows indicate the direction of the depicted relationships, whereas downward arrows denote decreased expression or activity. Dotted lines delineate different mechanistic modules and do not represent biological interactions.

As summarized in Figure 2, the anti-*H. pylori* actions of these alkaloids can be organized into several mechanistically distinct modules. First, COP and EPI exert direct antibacterial effects associated with disruption of bacterial structural integrity and fragmentation, whereas structurally optimized protoberberine derivatives provide additional evidence that membrane permeability and bacterial DNA are potentially druggable targets. Second, BBR, COP, EPI, and PAL interfere with the urease system at different levels, including catalytic activity, structural-gene expression, and accessory-protein-dependent maturation. Third, COP modulates bacterial virulence and stress-adaptation programs, including CagA- and *KatA*-associated processes. Finally, BBR may modify antibiotic susceptibility partly through regulation of the *hefA* efflux system, while BBR-containing regimens have shown adjunctive efficacy in clinical eradication studies. Delivery-system-based approaches may further enhance activity against bacterial biofilms. Importantly, these mechanisms differ substantially in their level of evidence and should not be considered uniformly established for all five alkaloids.

## 3. Suppression of Gastric Inflammatory Signaling and Remodeling of the Mucosal Immune Microenvironment

Chronic *H. pylori* infection induces persistent activation of inflammatory signaling pathways, which constitutes a major driving force linking sustained mucosal inflammation to gastric carcinogenesis. Among these pathways, the NF-κB and MAPK pathways play central roles in maintaining inflammatory responses, promoting cytokine production, and aggravating gastric mucosal injury. Available experimental evidence indicates that individual alkaloids regulate distinct but partially convergent inflammatory signaling nodes, thereby attenuating the inflammatory microenvironment associated with gastric mucosal damage and disease progression.

### 3.1. Inhibition of Pro-Inflammatory Signaling Pathways and Cytokine Responses

Following adhesion of *H. pylori* to gastric mucosal and glandular epithelial cells, host epithelial cells activate intracellular signaling networks in response to infection [76]. Persistent activation of these pathways promotes the transcription of inflammation-related genes and contributes to sustained gastric mucosal inflammation. Among them, NF-κB represents a central signaling pathway in *H. pylori*-associated gastritis [77,78]. Activation of NF-κB and related inflammatory networks promotes the expression of cytokines and chemokines, among which IL-8 represents an important mediator of *H. pylori* virulence factor-induced inflammatory responses [79].

Direct experimental evidence supports the ability of COP to regulate major intracellular inflammatory signaling pathways. In LPS-stimulated RAW264.7 macrophages, COP suppressed IκBα degradation and inhibited the phosphorylation of ERK, JNK, p38 MAPK, and PI3K/Akt. These signaling alterations were accompanied by decreased iNOS protein and mRNA expression, reduced NO production, and suppression of the pro-inflammatory cytokines IL-1β and IL-6 [80]. Notably, COP did not significantly affect TLR4 or MyD88 expression, suggesting that its anti-inflammatory activity primarily involves modulation of downstream NF-κB, MAPK, and PI3K/Akt signaling rather than inhibition of the proximal LPS–TLR4 recognition system [80].

Consistent with these findings, coptisine free base (CFB) suppressed NF-κB activation in experimental inflammatory models by reducing phosphorylation of IKKα/β and IκBα and nuclear accumulation of p65, while inhibiting p38 and JNK phosphorylation [43]. Because these experiments were not conducted in *H. pylori*-infected gastric models, they should be interpreted as supportive evidence for the intrinsic anti-inflammatory properties of the COP scaffold rather than as direct evidence of *H. pylori*-specific pathway regulation. More disease-specific evidence has been obtained for JAT. In an *H. pylori*-induced CAG rat model, JAT attenuated gastric inflammatory infiltration and mucosal pathological injury, reduced inflammatory cytokine levels, and suppressed *H. pylori* colonization. Mechanistically, JAT inhibited activation of NF-κB signaling and the NLRP3 inflammasome, supporting direct regulation of an inflammation-associated signaling axis in an *H. pylori*-related gastric disease model [81]. BBR also modulates *H. pylori*-associated inflammatory responses through several complementary mechanisms. In experimental *H. pylori*-associated gastritis models, BBR decreases the expression of pro-inflammatory mediators, including IL-6, TGF-β, and IL-1β, while increasing the anti-inflammatory cytokine IL-10 and attenuating BAFF-associated Th17 responses [39]. In addition, BBR regulates the IRF8–IFN-γ signaling axis in *H. pylori*-induced CAG. BBR treatment suppresses multiple IRF8/IFN-γ-associated inflammatory genes and proteins and alleviates *H. pylori*-induced gastric epithelial injury [82,83]. PAL acts through a distinct inflammation-associated signaling mechanism. MMP-mediated extracellular-matrix remodeling contributes to inflammatory infiltration and mucosal injury during chronic gastric inflammation [84]. In *H. pylori*-induced CAG models, PAL inhibited expression of ADAM17 and the EGFR ligand HB-EGF, thereby suppressing ADAM17/EGFR signaling and reducing MMP-10 expression [85]. These changes were accompanied by decreased CXCL16 and IL-8 expression, reduced inflammatory-cell infiltration, and improvement of gastric mucosal injury [85].

The NOS/NO system represents another component of alkaloid-mediated inflammatory regulation. *H. pylori*-associated chronic gastritis is characterized by increased inflammatory-cell infiltration and enhanced iNOS expression in gastric mucosal macrophages and lymphocytes [86]. Excessive iNOS-derived NO production may contribute to nitrosative stress and mucosal tissue injury. In LPS-stimulated RAW264.7 macrophages, COP suppressed both iNOS protein and mRNA expression and consequently reduced NO production [80]. Regulation of different NOS isoforms, however, appears to be context-dependent. In an ethanol-induced gastric injury model, BBR increased eNOS mRNA expression while suppressing iNOS mRNA expression [87]. Because the latter finding was obtained in a non-*H. pylori* gastric injury model, it should be interpreted as supportive evidence for modulation of the NOS/NO system rather than as a direct *H. pylori*-specific mechanism.

### 3.2. Remodeling of the Gastric Mucosal Immune Microenvironment

Persistent *H. pylori* infection disrupts the gastric mucosal immune microenvironment and contributes to the progression from chronic inflammation to precancerous lesions [88]. *H. pylori* disrupts the gastric mucosal immune barrier through its virulence factors, leading to hyperactivation of innate immune cells and dysfunction of adaptive immune responses. Alkaloids can modulate the phenotype and function of immune cells, thereby reshaping the immune homeostasis of the gastric mucosa.

Macrophage polarization represents an important component of *H. pylori*-associated mucosal immune remodeling. *H. pylori* infection promotes oxidative stress and can enhance pro-inflammatory M1-like macrophage responses [89]. The polarization pattern is influenced by infection intensity and other microenvironmental factors; for example, different multiplicities of infection can differentially affect M1- and M2-associated phenotypes [90]. In *H. pylori*-induced CAG, BBR regulates macrophage polarization through the IL-4/STAT6 signaling pathway and alters the expression of macrophage-associated inflammatory markers [91]. BBR-associated interventions also attenuate BAFF-induced Th17 responses [39,92]. Importantly, the functional significance of macrophage polarization is stage-dependent: attenuation of excessive M1-like inflammation may facilitate mucosal resolution during chronic gastritis, whereas M2-like tumor-associated macrophages in established gastric cancer can contribute to immunosuppression and tumor progression. Therefore, macrophage polarization should not be interpreted simply as an M1-to-M2 therapeutic shift across all stages of gastric carcinogenesis.

**Figure 3:**
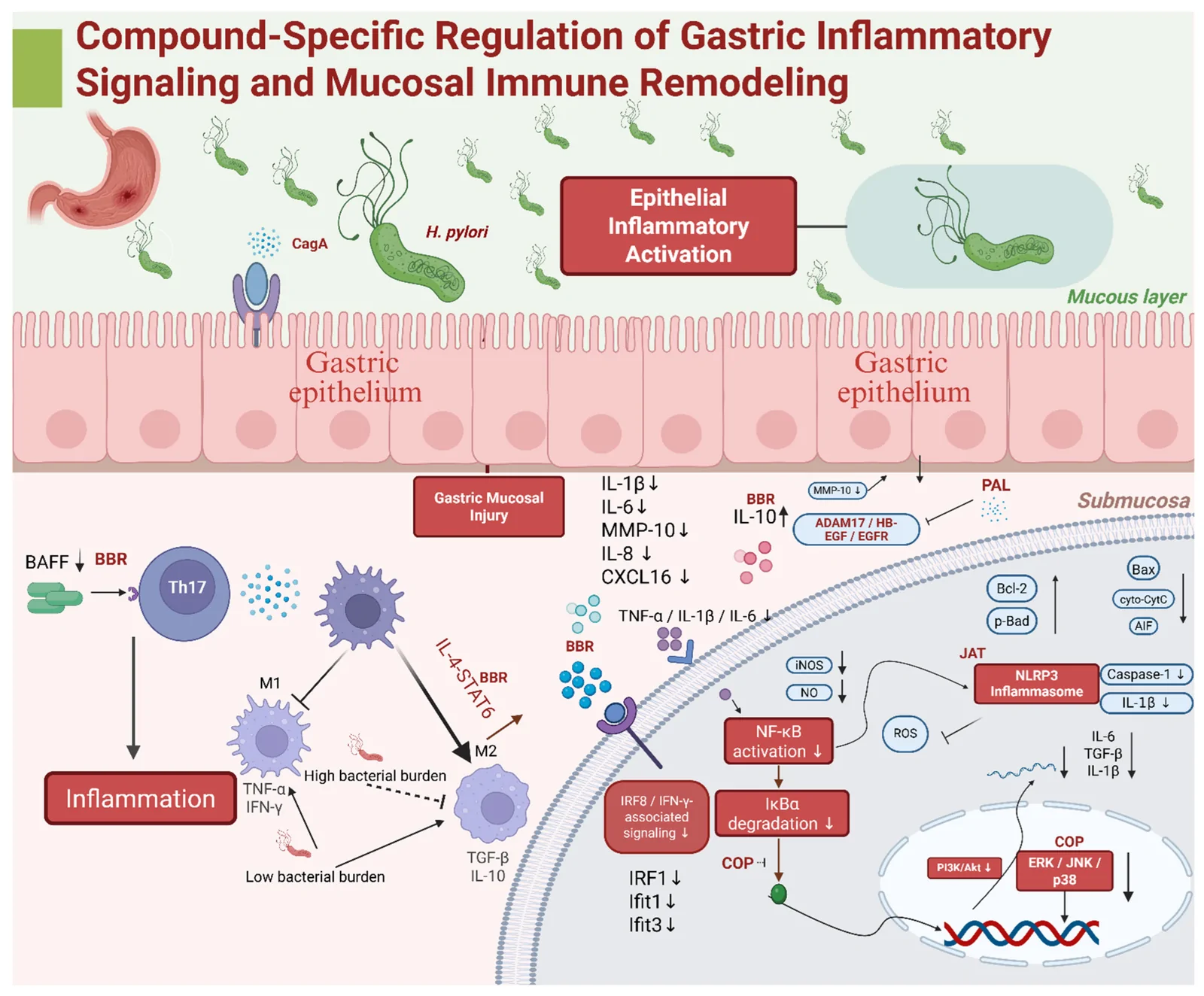
Compound-specific regulation of gastric inflammatory signaling and mucosal immune remodeling. COP suppresses NF-κB-, MAPK-, PI3K/Akt-, and iNOS/NO-associated inflammatory responses. JAT inhibits the NF-κB/NLRP3 inflammasome axis in *H. pylori*-associated gastric inflammation. PAL attenuates inflammatory remodeling through regulation of the ADAM17/HB-EGF/EGFR–MMP-10 pathway and downstream inflammatory mediators. BBR modulates IRF8/IFN-γ-associated signaling, BAFF/Th17 responses, and IL-4/STAT6-associated macrophage states. The functional consequences of macrophage polarization are context-dependent, and the illustrated mechanisms should not be interpreted as uniformly shared by all five alkaloids. Solid arrows indicate directional regulatory relationships, inhibitory lines indicate suppression, dashed lines denote indirect or context-dependent associations, and ↑/↓ indicate increased/decreased expression or activity.

As summarized in Figure 3, the anti-inflammatory and immunomodulatory actions of these alkaloids involve distinct but partially convergent regulatory networks. COP suppresses NF-κB-, MAPK-, PI3K/Akt-, and iNOS/NO-associated inflammatory responses; JAT inhibits the NF-κB/NLRP3 inflammasome axis in *H. pylori*-associated gastric inflammation; PAL modulates the ADAM17/EGFR/MMP-10 pathway; and BBR regulates IRF8/IFN-γ signaling as well as IL-4/STAT6- and BAFF/Th17-associated immune responses. These findings indicate that individual alkaloids act at different levels of the gastric inflammatory network rather than through a single uniformly shared pathway.

## 4. Preclinical Effects of Alkaloids on Gastric Precancerous Lesions: Atrophy, Intestinal Metaplasia, and Dysplasia

### 4.1. Amelioration of Gastric Glandular Atrophy and Mucosal Injury

Gastric glandular atrophy represents a major histopathological component of the precancerous gastric mucosal continuum. CAG is characterized by the loss of appropriate gastric glands and is closely associated with persistent *H. pylori*-induced inflammation or autoimmune gastric injury [93]. Alkaloids have long been reported to exhibit gastroprotective and mucosal protective activities [94], and accumulating preclinical evidence suggests that several protoberberine alkaloids can ameliorate glandular atrophy and associated gastric mucosal injury through distinct mechanisms.

BBR has been investigated in several experimental models of gastric mucosal injury and CAG. In *H. pylori*-injured GES-1 gastric epithelial cells, BBR improved cell viability and cellular morphology and reduced epithelial cell death [83]. A network-pharmacology and experimental study in CAG models further identified BBR as an important bioactive component associated with regulation of inflammation- and cell-survival-related signaling networks [95]. More recent multi-omics analyses demonstrated that BBR ameliorated gastric mucosal pathological injury through coordinated regulation of MAPK signaling, metabolic disturbances, and gut microbiota dysbiosis. In particular, BBR altered the relative abundance of microbial taxa including unclassified Muribaculaceae and Lactobacillus johnsonii, suggesting that microbiota–host metabolic interactions may contribute to its mucosal protective effects [96].

PAL also shows protective activity in experimental CAG. In an *H. pylori*-induced CAG rat model, PAL improved gastric mucosal histopathology and epithelial barrier integrity and modulated multiple metabolic abnormalities [97]. Metabolomic analysis implicated taurine and hypotaurine metabolism, glycerophospholipid metabolism, pentose and glucuronate interconversions, and interconnected energy-metabolic pathways in the protective response [97]. Earlier pharmacological studies also reported that PAL attenuated *H. pylori*-associated gastric mucosal injury [98].

JAT has also shown protective effects against gastric glandular injury. As discussed in Section 3.1, JAT attenuated *H. pylori*-associated gastric inflammation and mucosal pathological damage, an effect associated with inhibition of NF-κB/NLRP3 signaling [81]. In an MNNG-induced CAG rat model, JAT reduced gastric glandular disorganization and inflammatory infiltration and decreased mucosal cell death [99]. Mechanistically, JAT suppressed pyroptosis-associated proteins, including NLRP3, GSDMD, Caspase-1, and IL-1β. JAT also increased the anti-apoptotic protein Bcl-2 while reducing Bax and cleaved Caspase-3, indicating that coordinated inhibition of pyroptosis and excessive apoptosis may contribute to preservation of the gastric glandular compartment [99].

MNNG-based models are widely used in experimental studies of CAG and gastric precancerous injury and provide a chemically induced platform for investigating pharmacological modulation of glandular damage and inflammation [100,101]. Within MNNG-based CAG models, BBR has demonstrated reproducible mucosal protective effects. BBR treatment reduced inflammatory infiltration, improved glandular atrophy, and partially restored gastric physiological indices [100,102]. These effects have been associated with suppression of NF-κB and MAPK signaling and modulation of gut microbial composition [100]. A separate mechanistic study demonstrated that BBR regulated the TGF-β1/PI3K/Akt/mTOR signaling network, decreased inflammatory mediators, and increased autophagy-associated proteins including LC3-II and Beclin-1, suggesting that modulation of cell survival and autophagic responses may participate in gastric mucosal protection [103].

EPI provides another mechanistically distinct example. In MNNG-induced CAG, EPI ameliorated gastric mucosal injury through modulation of the EGFR–IL-33 axis [48]. This mechanism is biologically relevant because IL-33 is markedly increased in CAG and can be induced by both *H. pylori*-associated and chemical injury. Recent experimental studies further indicate that IL-33 promotes CAG progression by activating the AMPK–ULK1 autophagy pathway and accelerating degradation of the gastric mucosal protective protein GKN1 [104]. IL-33 has also been implicated in inflammatory myeloid-cell recruitment and gastric tumor-promoting microenvironmental remodeling [105,106]. Thus, suppression of aberrant IL-33 signaling may represent a potential mechanism linking EPI-mediated anti-inflammatory activity with preservation of gastric mucosal integrity.

PAL has additionally been shown to attenuate MNNG-induced CAG through the STAT1/CXCL10 axis. Comparative evaluation of five related alkaloids identified PAL as a particularly active compound in this experimental context. PAL reduced oxidative stress and inflammatory signaling, including IL-17, TNF-α, and p-p65, and these effects were associated with modulation of STAT1/CXCL10 signaling and improvement of gastric mucosal pathological injury [107].

### 4.2. Modulation of Intestinal Metaplasia, Dysplasia, and Precancerous Progression

Compared with the relatively extensive evidence for improvement of gastric glandular atrophy, direct evidence that individual alkaloids reverse established IM or Dys remains more limited. Nevertheless, several preclinical studies suggest that alkaloid-based interventions may modulate molecular processes associated with progression of gastric precancerous lesions. BBR-centered evidence has implicated regulation of cell-cycle and apoptosis-associated proteins, including Bcl-2, p53, and survivin, as well as modulation of autophagy-related pathways involving mTOR, LC3-II, and Beclin-1 [42]. These processes may influence epithelial survival and remodeling during progression from chronic mucosal injury toward metaplastic or dysplastic states, although molecular changes alone should not be interpreted as evidence of histological reversal. More direct evidence has been obtained from experimental gastric precancerous-lesion models in which BBR was evaluated alongside a multicomponent preparation. Network pharmacology identified AKT/HIF-1α/VEGF signaling as a candidate regulatory pathway, and subsequent animal experiments showed that both interventions reduced p-AKT, HIF-1α, and VEGF expression in gastric mucosal tissues, accompanied by improvement of glandular atrophy and potential intestinal metaplastic change [108]. These findings suggest that modulation of hypoxia- and angiogenesis-associated signaling may contribute to attenuation of precancerous mucosal progression.

**Figure 4:**
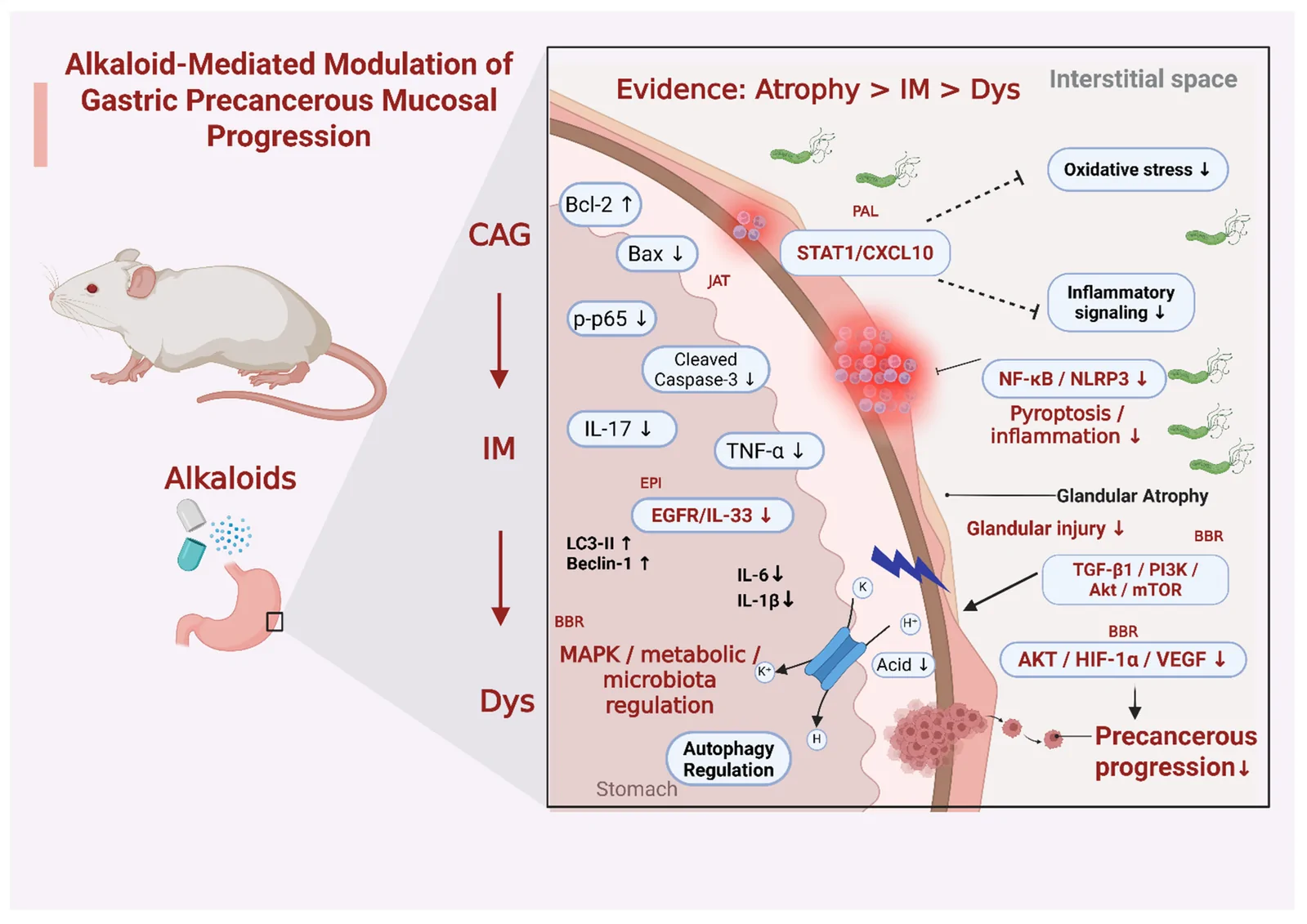
Alkaloid-mediated modulation of gastric precancerous mucosal progression. BBR, JAT, EPI, and PAL regulate distinct signaling networks associated with gastric glandular injury and progression of precancerous mucosal changes. BBR modulates TGF-β1/PI3K/Akt/mTOR-associated autophagic responses and AKT/HIF-1α/VEGF signaling; JAT suppresses NF-κB/NLRP3-associated pyroptosis and regulates Bcl-2/Bax/caspase-dependent apoptosis; EPI modulates the EGFR–IL-33 axis; and PAL attenuates STAT1/CXCL10-associated oxidative and inflammatory signaling. Current evidence is strongest for CAG and glandular atrophy, whereas direct evidence for established intestinal metaplasia and especially dysplasia remains more limited. Therefore, the illustrated effects represent amelioration of precancerous mucosal injury and attenuation of pathological progression rather than confirmed histological reversal. Arrows indicate directional regulatory associations, dotted lines indicate indirect or context-dependent relationships, and ↑/↓ indicate increased or decreased expression or activity.

Current evidence is strongest for CAG and glandular atrophy, whereas direct evidence for established IM or Dys remains limited and largely preclinical. These studies therefore support attenuation of precancerous mucosal injury rather than histological reversal of established lesions. The stage-specific preclinical evidence is summarized in Figure 4. Once malignant transformation has occurred, the therapeutic objective shifts from modulation of precancerous mucosal remodeling toward direct control of established tumor phenotypes, which is discussed in Section 5.

## 5. Multifaceted Antitumor Effects of Alkaloids in Established Gastric Cancer

### 5.1. Inhibition of Gastric Cancer Cell Proliferation and Induction of Apoptosis

**Figure 5:**
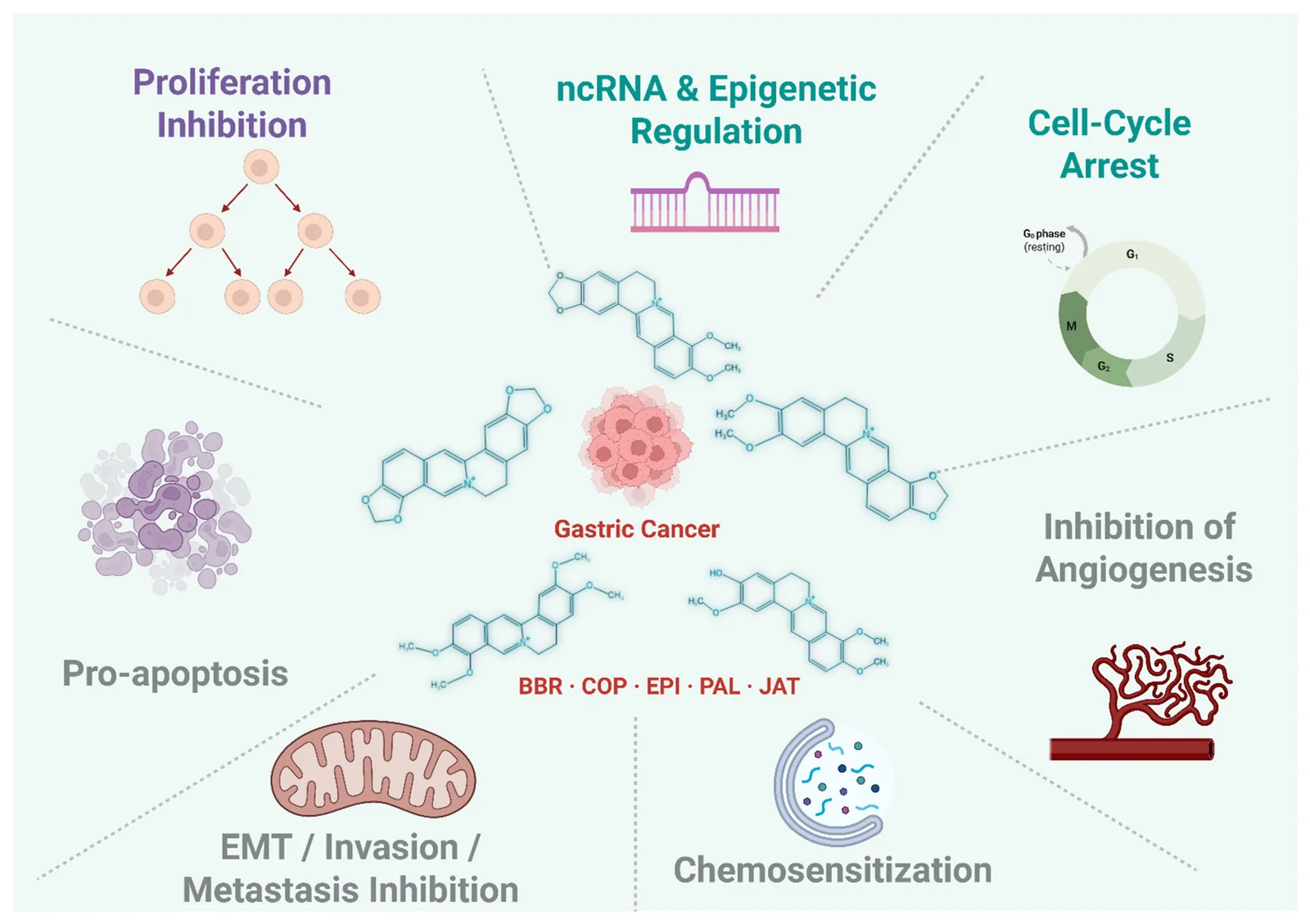
Antitumor and adjunctive effects of alkaloids in established gastric cancer. Preclinical studies indicate that protoberberine alkaloids regulate multiple malignant phenotypes, including tumor-cell proliferation, cell-cycle progression, apoptosis, EMT-associated invasion and metastasis, and angiogenic signaling. BBR-based interventions additionally regulate non-coding RNA and epigenetic networks and exhibit potential chemosensitizing activity toward selected cytotoxic agents. The strength of evidence differs substantially among individual compounds: BBR has the broadest mechanistic evidence, whereas direct evidence for COP, EPI, PAL, and JAT is more limited and stage specific. Chemosensitizing evidence is currently predominantly BBR based and remains preclinical.

The major antitumor phenotypes summarized across Section 5.1, Section 5.2, Section 5.3 and Section 5.4 are illustrated in Figure 5. Once malignant transformation has occurred, the pharmacological focus shifts from modulation of gastric mucosal injury toward direct control of established tumor-cell phenotypes. Available preclinical studies indicate that several alkaloids inhibit gastric cancer cell proliferation and promote programmed cell death, although the evidence is strongly dominated by BBR, with substantially fewer studies available for COP, EPI, PAL, and JAT.

BBR has been investigated extensively in gastric cancer cell and xenograft models. In BGC-823 gastric cancer cells, BBR reduced cell viability in a concentration- and time-dependent manner [109]. BBR and COP also inhibited proliferation of NCI-N87 cells in a dose-dependent manner [110]. In MGC-803 cells, BBR suppressed proliferation through modulation of MAPK signaling [111], whereas combined BBR and low-glucose treatment promoted apoptosis and suppressed malignant cellular activity through the PP2A/GSK3β/MCL-1 signaling pathway [112]. In xenograft models derived from MGC-803 and SGC-7901 cells, BBR delayed tumor growth in association with suppression of the HNF4α–WNT5a/β-catenin signaling pathway [113]. BBR also regulates several survival- and metabolism-associated signaling networks. In MKN-45 and HGC-27 gastric cancer cells, BBR inhibited IL-6/JAK2/STAT3 signaling, reduced p-JAK2, p-STAT3, Bcl-2, and Cyclin D1, and increased Bax and p21 expression, resulting in suppression of cell proliferation [114]. In MGC-803 cells, BBR-induced growth inhibition and apoptosis were additionally associated with downregulation of fatty acid-binding proteins and intracellular lipid accumulation, suggesting a link between altered lipid metabolism and tumor-cell death [115].

Cell-cycle regulation constitutes another major component of alkaloid-mediated antitumor activity. EPI directly interacts with GABRB3 in HGC-27 and MKN-45 cells, promotes p53 accumulation, activates the p21/CDK1/cyclin B1 axis, induces G2/M-phase arrest, and triggers apoptosis through the Bcl-2/Bax/caspase pathway [49]. In NCI-N87 cells, BBR, JAT, PAL, and COP increased the sub-G1 population and activated caspase-3/7, providing comparative evidence that multiple protoberberine alkaloids possess pro-apoptotic activity, although their potency differs [110]. BBR has also been reported to induce cell-cycle arrest and apoptosis in SGC-7901 and SNU-5 gastric cancer models [116,117]. Mitochondrial apoptosis represents an important downstream mechanism. In BBR-treated gastric cancer cells, increased Bax expression, decreased Bcl-2 expression, mitochondrial membrane-potential disruption, cytochrome-c release, and activation of caspase-3 and cleaved PARP have been observed [116,118]. Consistently, EPI treatment in MKN-45 xenograft tumors increased p53, p21, p27, Bax, cytochrome c, and cleaved caspase-3 while decreasing Bcl-2, consistent with activation of a p53-associated mitochondrial apoptotic pathway [50,119]. BBR and D-limonene have also shown combined antiproliferative activity in MGC-803 cells through cell-cycle perturbation, ROS generation, and mitochondrial apoptotic signaling [120].

Together, these studies indicate that alkaloids can interfere with established gastric cancer growth through partially convergent mechanisms involving survival signaling, metabolic regulation, cell-cycle checkpoints, and mitochondrial apoptosis. However, most available evidence remains derived from in vitro or xenograft models, and BBR accounts for the majority of mechanistic studies.

### 5.2. Suppression of Gastric Cancer Cell Invasion, Migration, and Metastasis

Invasion and metastasis are complex multistep processes involving epithelial–mesenchymal transition (EMT), extracellular-matrix (ECM) degradation, altered cell adhesion, and angiogenesis [121,122]. In gastric cancer, dysregulation of growth-factor signaling, including EGFR-associated pathways, contributes to aggressive tumor behavior [123,124]. *H. pylori* infection may further promote invasive phenotypes by inducing EMT-like changes and cancer stem cell-associated properties, particularly through CagA-dependent mechanisms [125,126]. Preclinical evidence indicates that BBR can interfere with several of these metastatic processes. In gastric cancer models, BBR suppressed HIF-1α and VEGF expression, indicating potential inhibition of hypoxia-associated angiogenic signaling [127]. In SNU-5 gastric cancer cells, BBR reduced MMP-1, MMP-2, and MMP-9 expression and inhibited cell migration, consistent with suppression of ECM-degrading and metastatic phenotypes [128]. EMT represents another important target of BBR. BBR increased epithelial-associated proteins, including E-cadherin and ZO-1, while decreasing mesenchymal markers such as N-cadherin and vimentin. Mechanistically, BBR interacted with TGFβR1 and TGFβR2 and suppressed TGF-β/Smad-associated signaling, with additional modulation of PI3K/Akt signaling, thereby attenuating EMT-associated invasive behavior in gastric carcinoma cells [129].

Taken together, these studies suggest that BBR can suppress invasion- and metastasis-associated phenotypes through regulation of hypoxia/angiogenesis, ECM degradation, and EMT. However, compared with the extensive BBR literature, direct evidence for COP, EPI, PAL, and JAT in gastric cancer invasion or metastasis remains sparse.

### 5.3. Regulation of Non-Coding RNA and Epigenetic Networks in Gastric Cancer

Non-coding RNAs and epigenetic alterations constitute an additional regulatory layer in gastric carcinogenesis by reshaping gene-expression programs and cancer-associated signaling networks [130]. *H. pylori* infection itself can remodel these regulatory systems. For example, CagA suppresses let-7 expression through epigenetic mechanisms involving histone modification and DNA methylation, thereby promoting Ras-associated signaling during *H. pylori*-related carcinogenesis [131]. miR-146a is also induced in *H. pylori*-infected gastric epithelial cells and participates in negative-feedback regulation of inflammatory responses, illustrating the context-dependent functions of individual miRNAs during *H. pylori*-associated gastric disease [132].

Emerging evidence suggests that BBR-based interventions can further alter miRNA regulatory networks in gastric cancer models. In BBR-treated SGC-7901 cells, changes in miRNA expression profiles were predicted to converge on multiple cancer-associated signaling pathways, including Hippo, Notch, and FoxO networks [117]. Because these pathway associations were derived primarily from integrated miRNA–mRNA profiling and pathway analysis, they should be interpreted as candidate regulatory networks rather than fully validated causal mechanisms. A more specific regulatory axis has been described using BBR-loaded chitosan/pectin nanoparticles in AGS gastric cancer cells. This formulation increased miR-185-5p expression and decreased *KLF7* mRNA expression, changes that were associated with reduced malignant cellular activity [133]. KLF7 has independently been implicated in regulation of gastric cancer proliferation [134], supporting the biological relevance of this regulatory node.

Epigenetic remodeling may also contribute to the antitumor effects of BBR-based formulations. Global DNA hypomethylation is associated with chromosomal instability and advanced *H. pylori*-associated gastric neoplasia [135]. In AGS cells, BBR-loaded nanoparticles increased global 5-methylcytosine levels and altered DNA methyltransferase expression, suggesting that modulation of DNA methylation may contribute to their cytotoxic and regulatory effects [133]. Importantly, these findings were obtained using a nanoparticle formulation and should not be assumed to represent identical effects of unformulated BBR.

Collectively, current evidence suggests that alkaloid-based interventions may influence gastric cancer through non-coding RNA and epigenetic regulatory networks. However, this area remains at an early stage, is dominated by BBR-based studies, and contains limited evidence for COP, EPI, PAL, or JAT.

### 5.4. Chemosensitization and Adjunctive Effects of Alkaloids

Chemoresistance and treatment-related toxicity remain important limitations of systemic therapy for gastric cancer. In addition to their direct antitumor activities, alkaloids may exert adjunctive effects by increasing tumor-cell sensitivity to conventional chemotherapeutic agents and by attenuating chemotherapy-associated gastrointestinal injury. Current evidence in this area is predominantly centered on BBR, whereas comparable studies of COP, EPI, PAL, and JAT remain scarce.

BBR has been reported to enhance cisplatin sensitivity through regulation of apoptosis and drug-resistance-associated signaling networks. In cisplatin-resistant gastric cancer cells, BBR increased miR-203 expression and reduced expression of its anti-apoptotic target Bcl-w, thereby promoting apoptosis and enhancing cellular responsiveness to cisplatin [136]. This regulatory axis is of particular interest because *H. pylori*-associated downregulation of miR-203 has been linked to enhanced gastric cancer cell proliferation and invasion [137]. Thus, restoration of the miR-203-associated tumor-suppressive network may contribute to the chemosensitizing effects of BBR. Additional evidence indicates that BBR-mediated cisplatin sensitization is not restricted to miRNA regulation. In cisplatin-resistant gastric cancer models, BBR decreased the expression of the drug-efflux proteins MDR1/P-glycoprotein and MRP1, enhanced caspase-associated apoptosis, and suppressed PI3K/AKT/mTOR signaling. Combined BBR–cisplatin treatment also enhanced tumor suppression in a resistant gastric cancer xenograft model [138]. These findings suggest that BBR may influence multiple components of the chemoresistant phenotype, including drug efflux, survival signaling, and apoptotic thresholds.

BBR has also demonstrated potential interactions with 5-fluorouracil (5-FU). In AGS gastric cancer cells, BBR reduced STAT3 activation and survivin expression and decreased cell viability. When combined with 5-FU, BBR produced stronger suppression of STAT3/survivin-associated prosurvival signaling and enhanced cancer-cell death compared with either treatment alone [139]. These findings indicate that inhibition of STAT3/survivin signaling may represent one mechanism through which BBR enhances the activity of 5-FU.

The potential adjunctive value of BBR may extend beyond tumor-cell chemosensitization to protection against chemotherapy-associated gastrointestinal injury. In a 5-FU-induced intestinal mucositis model, BBR reduced body-weight loss and diarrhea, preserved intestinal barrier integrity, increased occludin expression, and decreased mucosal IL-1β, IL-6, and TNF-α levels [140]. These protective effects were accompanied by remodeling of the gut microbiota and fecal metabolic profile, including increased butyrate and glutamine levels. Fecal microbiota transplantation from BBR-treated animals partially reproduced the protective phenotype, supporting a contribution of microbiota–metabolite interactions to attenuation of chemotherapy-induced mucosal injury [140]. More recent formulation-based studies provide proof-of-concept that enhancement of antitumor efficacy and mitigation of treatment-associated toxicity may potentially be integrated. An oral BBR nanocapsule platform designed for prolonged gastrointestinal retention potentiated oxaliplatin-mediated tumor suppression in preclinical gastric cancer models while protecting intestinal mucosal integrity and reducing treatment-associated systemic toxicity [141]. These effects were accompanied by remodeling of the microbiota–immune axis, suggesting that drug-delivery optimization may expand the adjunctive potential of BBR [141].

**Figure 6:**
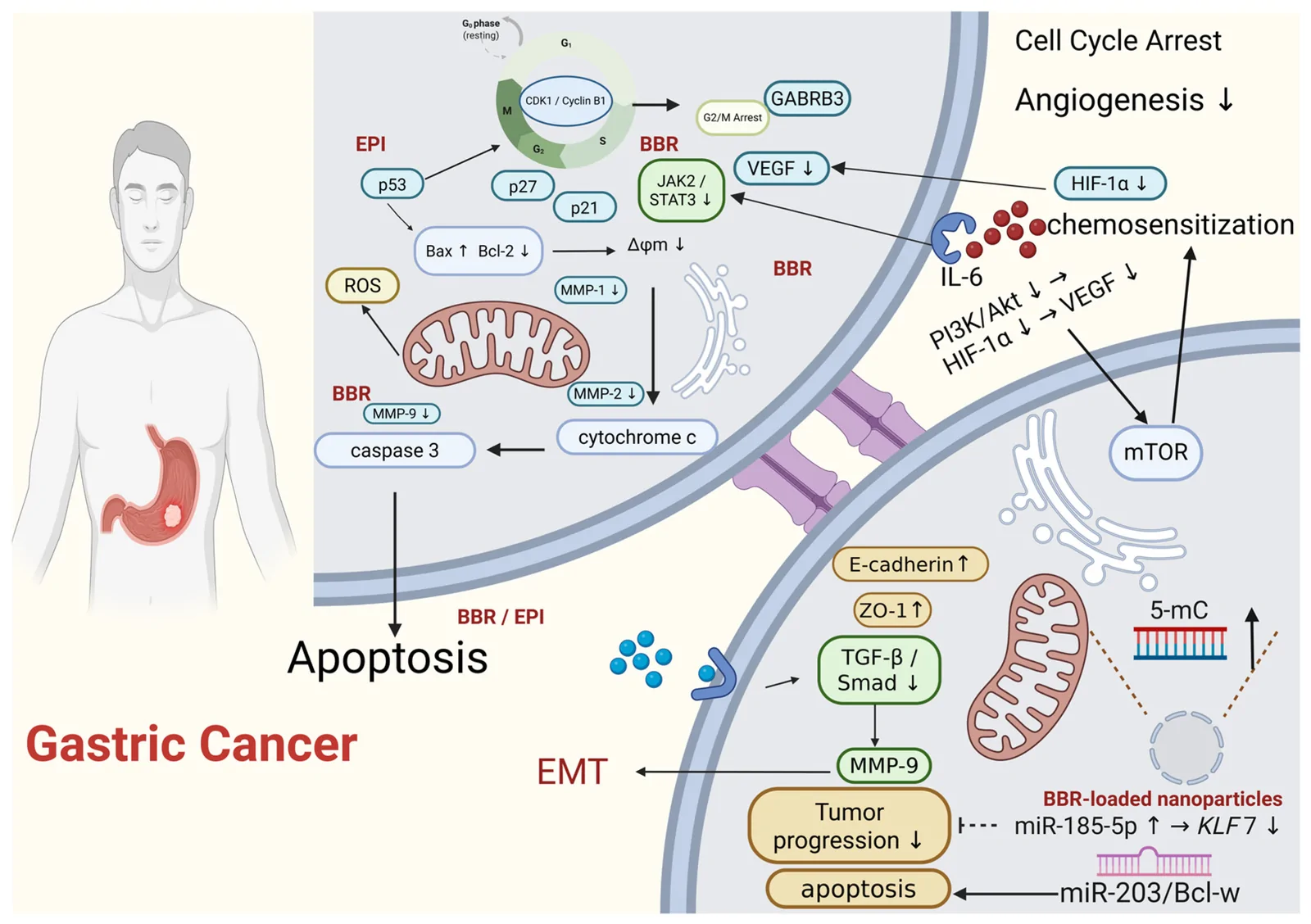
Molecular networks underlying the antitumor and chemosensitizing effects of alkaloids in gastric cancer. The figure summarizes major molecular mechanisms associated with alkaloid treatment in established gastric cancer. EPI regulates GABRB3-associated p53 accumulation and the p21/CDK1/cyclin B1 axis, contributing to G2/M cell-cycle arrest and mitochondrial apoptosis. BBR-dominated evidence indicates regulation of IL-6/JAK2/STAT3 signaling, mitochondrial apoptotic pathways, HIF-1α/VEGF-associated angiogenesis, MMP-mediated extracellular-matrix degradation, and TGF-β/Smad- and PI3K/Akt-associated EMT. BBR-based studies further suggest regulation of non-coding RNA and epigenetic networks, including formulation-dependent miR-185-5p/KLF7 and DNA-methylation changes, as well as potential chemosensitizing effects involving miR-203/Bcl-w, drug-efflux proteins, and PI3K/Akt/mTOR signaling. The illustrated mechanisms are predominantly supported by preclinical studies, and evidence for COP, PAL, and JAT in established gastric cancer remains comparatively limited. Arrows indicate directional regulatory relationships, inhibitory lines indicate suppression, dotted lines indicate indirect or association-based relationships, and ↑/↓ denote increased/decreased expression or activity.

Taken together, current evidence suggests two potentially complementary adjunctive roles for BBR in gastric cancer therapy: enhancement of tumor sensitivity to cytotoxic agents and attenuation of chemotherapy-associated gastrointestinal injury. These effects involve distinct but interconnected mechanisms, including miRNA regulation, suppression of drug-efflux proteins, inhibition of PI3K/AKT/mTOR and STAT3/survivin signaling, promotion of apoptosis, and modulation of the intestinal microbiota–immune axis. Nevertheless, these findings are derived predominantly from cell and animal models and should not be interpreted as evidence that BBR currently reverses chemoresistance or reduces chemotherapy dosage requirements in patients with gastric cancer. Furthermore, comparable chemosensitizing evidence for COP, EPI, PAL, and JAT remains largely unavailable. Future studies should therefore prioritize pharmacokinetically achievable concentrations, clinically relevant experimental models, standardized combination regimens, and prospective clinical evaluation. The integrated antitumor and chemosensitizing networks are summarized in Figure 6.

## 6. Discussion

This review integrates the available evidence on BBR, COP, EPI, PAL, and JAT across the continuum of gastric carcinogenesis, encompassing *H. pylori* infection, chronic gastric inflammation, precancerous mucosal changes, and established gastric cancer. Rather than representing pharmacologically equivalent compounds, the five alkaloids exhibit markedly different evidence profiles, with BBR currently having the broadest evidence base across multiple disease stages. Based on the pathological sequence of gastric carcinogenesis and the evidence summarized in Section 2, Section 3, Section 4 and Section 5, their reported actions can be organized into a three-tier intervention framework: (I) etiologic and inflammatory interception, (II) modulation of precancerous mucosal progression, and (III) antitumor and adjunctive intervention in established gastric cancer.

The first tier, etiologic and inflammatory interception, encompasses both suppression of *H. pylori* persistence and attenuation of infection-driven gastric inflammation. At the bacterial level, individual alkaloids act through partially distinct mechanisms, including direct bacterial injury, inhibition of urease-dependent acid adaptation, modulation of bacterial virulence and stress-associated processes, and alteration of antibiotic susceptibility. BBR may partially modulate antibiotic susceptibility through regulation of the *hefA* efflux system, whereas COP and EPI exhibit more direct evidence of bacterial structural injury. BBR, COP, EPI, and PAL also interfere with different components of the urease system. These mechanisms indicate that the anti-*H. pylori* effects of protoberberine alkaloids are distributed across several bacterial vulnerabilities rather than being attributable to a single antibacterial target.

At the host level, alkaloids regulate several inflammatory and immune pathways associated with *H. pylori*-driven gastric injury. BBR attenuates BAFF-associated Th17 responses in *H. pylori*-infected chronic gastritis models [39]. JAT suppresses NF-κB activation and NLRP3 inflammasome-associated inflammatory responses [81], whereas PAL modulates oxidative and inflammatory injury through the STAT1/CXCL10 axis [107]. EPI acts through a distinct mechanism involving regulation of the EGFR–IL-33 axis [48]. Together, these findings suggest that the five alkaloids provide partially complementary regulation of bacterial and host inflammatory processes; however, the specific mechanisms and strength of evidence differ among compounds and should not be interpreted as uniformly established properties of the entire alkaloid class.

The second tier, modulation of precancerous mucosal progression, concerns the potential of alkaloids to ameliorate gastric glandular injury and regulate biological processes associated with progression toward IM and Dys. Among the histopathological stages of the Correa cascade, the most consistent pharmacological evidence currently concerns CAG and gastric glandular atrophy. Several alkaloids regulate inflammatory signaling, programmed cell death, autophagy, metabolic homeostasis, and microbiota-associated processes in experimental models of gastric mucosal injury. For example, BBR modulates the TGF-β1/PI3K/Akt/mTOR network together with autophagy-associated proteins in CAG models [103]. Studies involving BBR and a multicomponent intervention implicate the AKT/HIF-1α/VEGF axis in the regulation of gastric precancerous mucosal changes [108].

These findings suggest that alkaloids may influence both epithelial injury and the microenvironmental conditions that support progression of gastric precancerous lesions. Nevertheless, the evidence is substantially stronger for CAG and glandular atrophy than for established IM or Dys. Molecular changes in apoptosis-, autophagy-, hypoxia-, or angiogenesis-associated pathways should therefore not be interpreted as direct evidence of histological reversal. At present, the available data are more appropriately viewed as evidence for amelioration of precancerous mucosal injury and attenuation of pathological progression rather than definitive reversal of established gastric precancerous lesions.

The third tier, antitumor and adjunctive intervention in established gastric cancer, encompasses both direct regulation of malignant tumor phenotypes and potential enhancement of conventional chemotherapy. Preclinical studies indicate that alkaloids can suppress gastric cancer cell proliferation, induce cell-cycle arrest and apoptosis, and interfere with invasion-, EMT-, angiogenesis-, and metastasis-associated pathways. However, evidence at this stage is strongly dominated by BBR, whereas direct gastric cancer evidence for COP, EPI, PAL, and JAT remains considerably more limited. A comparative stage-specific evidence profile of the five alkaloids is summarized in Table 2.

BBR-centered studies additionally indicate potential chemosensitizing effects. As discussed in Section 5.4, BBR may enhance cisplatin responsiveness through regulation of the miR-203/Bcl-w axis and drug-efflux-associated mechanisms, while suppression of PI3K/AKT/mTOR and STAT3/survivin signaling may contribute to enhanced responsiveness to selected cytotoxic agents. Preclinical studies further suggest that BBR may attenuate chemotherapy-associated gastrointestinal mucosal injury. These findings support investigation of BBR as a potential adjunctive agent; however, they should not be interpreted as evidence that BBR or the other four alkaloids currently reverse chemotherapy resistance or permit reduction in chemotherapy dosage in patients with gastric cancer.

Collectively, these mechanistic observations indicate that the five alkaloids form a partially overlapping rather than completely shared pharmacological network. Their actions extend from bacterial and inflammatory regulation to modulation of epithelial survival, metabolic homeostasis, tissue remodeling, and established tumor phenotypes. This mechanistic diversity provides a biological rationale for the proposed three-tier framework, but it also highlights a central limitation of the current literature: evidence density is highly uneven among compounds and disease stages. BBR has been examined across almost the entire gastric carcinogenesis continuum, whereas COP, EPI, PAL, and particularly JAT are supported by substantially fewer stage-specific studies. Accordingly, the current evidence favors a stage-oriented, compound-specific research strategy rather than interchangeable use of the five alkaloids. Combination approaches remain exploratory and should be compared directly with the corresponding single compounds using chemically defined preparations before any preferred multi-alkaloid regimen can be proposed.

Despite substantial progress in antibacterial research, important gaps remain regarding *H. pylori* adhesion, persistence, strain heterogeneity, and long-term host–pathogen interactions. Recent BBR derivatives with activity against multidrug-resistant *H. pylori* illustrate the optimization potential of the protoberberine scaffold [142], although effects of modified derivatives should not be directly attributed to native BBR. In addition, persistent alterations in the gastric microbiota after *H. pylori* eradication have been associated with inflammation, glandular atrophy, and intestinal metaplasia [143], but whether alkaloids modulate this broader microbial ecosystem remains unclear.

Evaluation of gastric precancerous lesions also remains largely dependent on conventional histopathological endpoints. Injury-associated epithelial-state transitions, including SPEM, have received comparatively limited attention [144,145,146,147], and gastric carcinogenesis should not be simplified into a single linear lineage sequence. Lineage tracing, organoid models, and single-cell or spatial approaches may help distinguish attenuation of tissue injury from restoration of normal epithelial differentiation [148]. Future clinical studies may preferentially evaluate high-risk individuals with persistent advanced atrophy and/or intestinal metaplasia after successful *H. pylori* eradication, although this should be regarded as a trial-enrichment strategy rather than an established indication. This population may include older individuals with persistent, extensive, or histologically advanced atrophy and/or intestinal metaplasia after successful *H. pylori* eradication. The optimal compound, dose, treatment duration, long-term safety, and cost-effectiveness remain undefined and require pharmacokinetic, dose-finding, long-term safety, and prospective clinical studies.

In established gastric cancer, most studies remain focused on proliferation and apoptosis, whereas invasion, treatment resistance, and tumor-microenvironmental remodeling are less comprehensively investigated. These processes are strongly influenced by the tumor immune microenvironment [149], including tumor-associated macrophages [150], cancer-associated fibroblasts [151], myeloid-derived suppressor cells, and cytotoxic T-cell states [152,153], while *H. pylori*-associated metabolic reprogramming may provide an additional link between infection and tumor biology [154]. Future studies should therefore incorporate more clinically relevant multicellular and patient-derived models. Species differences and the use of legacy misidentified cell lines such as BGC-823 and SGC-7901 should also be considered when extrapolating preclinical findings to human disease.

Drug-delivery and pharmacokinetic constraints represent major translational challenges. BBR provides a particularly well-characterized example of this exposure limitation. Preclinical pharmacokinetic studies have demonstrated very low systemic exposure after oral administration, with extensive intestinal first-pass elimination contributing substantially to its low plasma concentrations [155]. P-glycoprotein-mediated intestinal efflux has also been identified as an important factor limiting BBR absorption [156]. These pharmacokinetic characteristics indicate that pharmacological effects observed at relatively high in vitro concentrations should not be assumed to correspond directly to equivalent systemic concentrations achievable in vivo. Experimental absorption studies have also demonstrated limited intestinal permeability and absorption of COP, PAL, and JAT [157], while EPI has been evaluated separately with respect to oral bioavailability, excretion, and CYP450 inhibition [158]. Nevertheless, the available pharmacokinetic evidence remains uneven across BBR, COP, EPI, PAL, and JAT and is not sufficiently standardized to support reliable cross-compound comparisons of oral bioavailability, plasma exposure, metabolic disposition, or gastric mucosal distribution.

The pharmacological performance of alkaloids can be influenced by poor solubility, limited systemic exposure, gastric luminal conditions, mucus penetration, and *H. pylori* biofilm formation. Advances in nanodelivery systems provide a potential strategy to overcome selected barriers [159]. BBR-loaded mannosylerythritol lipid-B nanomicelles have been reported to enhance activity against *H. pylori* biofilms in vivo, reduce bacterial burden, and improve gastric mucosal injury [160]. Self-assembled nanodrugs composed of lipophilic BBR derivatives and rhamnolipids can further enhance penetration of mucus and bacterial biofilm barriers [161]. More recently, a mucoadhesive lignin–liposome system loaded with COP demonstrated strong antibacterial activity against antibiotic-resistant *H. pylori* and substantial disruption of established biofilms [75]. These results support drug-delivery optimization as an important translational direction. Nevertheless, formulation-dependent effects should be distinguished from the intrinsic pharmacological activity of free alkaloids. Future studies should determine whether targeted, mucoadhesive, pH-responsive, or other stimuli-responsive delivery systems can achieve pharmacologically meaningful local or systemic exposure while maintaining acceptable safety and formulation reproducibility.

Combination strategies remain exploratory and require cautious interpretation. Several multicomponent preparations have provided indirect evidence relevant to combined intervention. In cancer-cell models, one such preparation suppressed cell growth through regulation of Cyclin B1/CDC2-associated cell-cycle signaling [162]. Activity has also been reported in experimental models of gastrointestinal inflammation [163], while a separate study demonstrated efficacy against drug-resistant *H. pylori* infection [164]. Another multicomponent intervention reduced *H. pylori* colonization and NLRP3-associated inflammatory responses [165]. In a rat model of gastric precancerous lesions, modulation of macrophage polarization was associated with improvement of mucosal pathology [166]. Gastroprotective effects against *H. pylori*-induced gastric epithelial injury have also been reported for a distinct multicomponent formulation [167]. However, because these preparations contain multiple pharmacologically active constituents, they do not provide direct evidence of synergy among purified BBR, COP, EPI, PAL, and JAT. These studies are therefore better regarded as indirect support for investigating combination strategies rather than as evidence for an established multi-alkaloid regimen. Direct evaluation of chemically defined alkaloid combinations should compare fixed-ratio combinations with the corresponding single compounds and incorporate quantitative synergy analysis, pharmacokinetic assessment, and standardized toxicity evaluation before any preferred combination can be proposed.

In summary, the available evidence supports a three-tier framework comprising etiologic and inflammatory interception, modulation of precancerous mucosal progression, and antitumor and adjunctive intervention in established gastric cancer. The value of this framework lies in organizing a heterogeneous body of evidence according to the pathological continuum of gastric carcinogenesis rather than implying that all five alkaloids exert equivalent effects at every disease stage. BBR currently has the broadest evidence base, whereas COP, EPI, PAL, and JAT show more stage-restricted and substantially less mature evidence profiles. Most evidence for gastric precancerous-lesion modulation and gastric cancer treatment remains preclinical. Future work integrating optimized drug-delivery systems, chemically defined combination strategies, epithelial lineage-resolved models, and multi-omics analyses may help clarify the pharmacological boundaries, optimal disease-stage positioning, and translational potential of protoberberine alkaloids.

## Figures and Tables

**Table 1:** Chemical Structures and Reported Pharmacological Activities of the Alkaloids Discussed in This Review.

Alkaloid	Molecular Formula	Structural Formula	Effect	References
BBR	C_20_H_18_NO_4_^+^	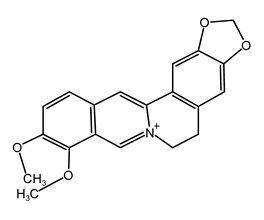	Antibacterial activity, anti-inflammatory, antitumor activity	[38,39,42]
COP	C_19_H_14_NO_4_^+^	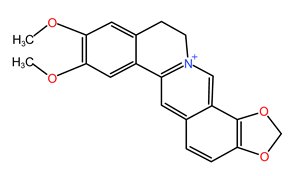	Antibacterial activity, anti-inflammatory activity, antitumor activity	[44]
EPI	C_20_H_18_NO_4_^+^	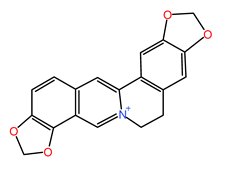	Anti-*H. pylori* and urease inhibition; amelioration of experimental CAG; antitumor activity	[46,47,48,49,50]
PAL	C_21_H_22_NO_4_^+^	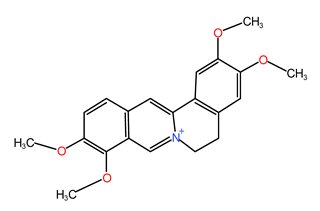	Antitumor activity, antioxidant activity, anti-inflammatory activity and microbiota-regulatory effects	[52,53,54,55]
JAT	C_20_H_20_NO_4_^+^	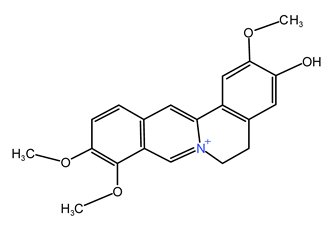	Antidiabetic activity, antilipidemic and anti-obesity effects, anti-rheumatoid arthritis, antimicrobial effect, antitumor activity, neuroprotective effect	[56]

**Table 2:** Stage-Specific Evidence Profile of the Five Alkaloids across Gastric Carcinogenesis.

Alkaloid	Tier I: Etiologic and Inflammatory Interception	Tier II: Modulation of Precancerous Mucosal Progression	Tier III: Antitumor and Adjunctive Intervention in Established Gastric Cancer	Overall Evidence Profile
BBR	Inhibition of the urease system; modulation of *hefA*-associated antibiotic susceptibility; adjunctive activity in *H. pylori* eradication regimens; regulation of BAFF/Th17- and IRF8/IFN-γ-associated inflammatory responses [39,65,69,71,72,73,74,82,83]	Amelioration of CAG and glandular atrophy; regulation of MAPK signaling, metabolic homeostasis, and gut microbiota; modulation of TGF-β1/PI3K/AKT/mTOR–autophagy and AKT/HIF-1α/VEGF-associated pathways [95,96,100,102,103,108]	Inhibition of proliferation; induction of cell-cycle arrest and mitochondrial apoptosis; suppression of EMT, migration, and invasion; regulation of non-coding RNA/epigenetic networks; preclinical chemosensitizing activity in cisplatin- and 5-FU-based treatment [109,111,112,113,114,115,116,117,118,127,128,129,133,136,139]	Broadest evidence base. Evidence spans all three tiers and includes limited human evidence for *H. pylori* eradication. Evidence for precancerous-lesion modulation and gastric cancer treatment remains predominantly preclinical.
COP	Direct bacterial structural injury; suppression of CagA-associated pathogenicity; inhibition of urease activity and UreG-dependent maturation; activity against clarithromycin-resistant *H. pylori*; regulation of NF-κB/MAPK/PI3K-Akt/iNOS-associated inflammatory signaling [59,66,70,80]	Direct evidence for purified COP in gastric precancerous-lesion models remains limited in the literature summarized in this review.	Antiproliferative and pro-apoptotic activity has been observed in comparative gastric cancer cell studies [110], but mechanistic evidence remains sparse.	Evidence is concentrated in anti-*H. pylori* and inflammatory mechanisms. Precancerous-lesion and established gastric cancer evidence is substantially less developed than for BBR.
EPI	Direct bacteriostatic/bactericidal activity and bacterial fragmentation; inhibition of urease activity and *ureB* expression [46,47]	Amelioration of experimental CAG through regulation of the EGFR–IL-33 axis [48]	Regulation of GABRB3-associated p53 accumulation; p21/CDK1/cyclin B1-mediated G2/M arrest; induction of mitochondrial apoptosis; antitumor activity in gastric cancer xenograft models [49,50]	Moderate but exclusively preclinical evidence spanning all three tiers, with relatively clear mechanistic evidence for urease inhibition and p53-dependent tumor-cell regulation.
PAL	Inhibition of *H. pylori* urease; regulation of ADAM17/EGFR/MMP-10-associated inflammatory remodeling [58,67,85]	Improvement of experimental CAG; modulation of metabolic abnormalities and STAT1/CXCL10-associated oxidative and inflammatory signaling [97,98,107]	Pro-apoptotic activity has been observed in comparative gastric cancer cell studies [110], but mechanistic evidence in established gastric cancer remains limited.	Evidence is concentrated in gastric inflammation and CAG. Direct evidence for established gastric cancer is substantially less extensive.
JAT	Suppression of *H. pylori*-associated inflammation and colonization through regulation of NF-κB/NLRP3 inflammasome signaling [81]	Amelioration of MNNG-induced CAG through regulation of pyroptosis- and apoptosis-associated pathways, including NLRP3/GSDMD/Caspase-1 and Bcl-2/Bax/Caspase-3 [99]	Limited comparative evidence indicates pro-apoptotic activity in gastric cancer cells [110].	Sparse and predominantly preclinical evidence. Current studies are concentrated mainly on *H. pylori*-associated inflammation and CAG.

## Data Availability

The data presented in this study are available on request from the corresponding authors. We confirm that all figures in this manuscript are original content created by the authors using BioRender. We hold a valid commercial license for this software, which grants full publication rights for all content created with it.

## Abbreviations

**Abbreviation**	**Full Term**
5-FU	5-fluorouracil
ADAM17	ADAM metallopeptidase domain 17
Akt	protein kinase B
Bax	Bcl-2-associated X protein
BBR	berberine
Bcl-2	B-cell lymphoma-2
CAFs	cancer-associated fibroblasts
CAG	chronic atrophic gastritis
CagA	cytotoxin-associated gene A
Caspase	cysteinyl aspartate specific proteinase
CDX2	caudal type homeobox 2
CFB	coptisine free base
COP	coptisine
CXCL10	C-X-C motif chemokine ligand 10
Dys	dysplasia
EGFR	epidermal growth factor receptor
EMT	epithelial–mesenchymal transition
eNOS	endothelial nitric oxide synthase
EPI	epiberberine
*H. pylori*	*Helicobacter pylori*
HPU	*H. pylori* urease
HDI	Human Development Index
HIF-1α	hypoxia-inducible factor-1α
IFN-γ	interferon-γ
IL-1	interleukin 1
IL-6	interleukin 6
IL-10	interleukin 10
IM	intestinal metaplasia
iNOS	inducible nitric oxide synthase
JAK2	Janus kinase 2
JAT	jatrorrhizine
LC3	microtubule-associated protein 1 light chain 3
MAPK	mitogen-activated protein kinase
MDSCs	myeloid-derived suppressor cells
MIC	minimum inhibitory concentration
miRNA	microRNA
MMP	matrix metalloproteinase
MNNG	N-methyl-N’-nitro-N-nitrosoguanidine
mTOR	mammalian target of rapamycin
MUC2	mucin 2
MUC6	mucin 6
NF-κB	nuclear factor-kappa B
NLRP3	NLR family pyrin domain containing 3
p53	tumor protein p53
PAL	palmatine
PI3K	phosphatidylinositol 3-kinase
ROS	reactive oxygen species
SPEM	spasmolytic polypeptide-expressing metaplasia
STAT	signal transducer and activator of transcription
TAMs	tumor-associated macrophages
TFF2	trefoil factor 2
TGF-β	transforming growth factor-β
Th17	T helper 17 cell
TNF-α	tumor necrosis factor-α
VacA	vacuolating cytotoxin A
VEGF	vascular endothelial growth factor

## References

[1] Yang W.-J., Zhao H.-P., Yu Y., Wang J.-H., Guo L., Liu J.-Y., Pu J., Lv J. (2023). Updates on Global Epidemiology, Risk and Prognostic Factors of Gastric Cancer. World J. Gastroenterol..

[2] Sundar R., Nakayama I., Markar S.R., Shitara K., van Laarhoven H.W.M., Janjigian Y.Y., Smyth E.C. (2025). Gastric Cancer. Lancet.

[3] Li M., Cao S., Xu R.-H. (2025). Global Trends and Epidemiological Shifts in Gastrointestinal Cancers: Insights from the Past Four Decades. Cancer Commun..

[4] Ilic M., Ilic I. (2022). Epidemiology of Stomach Cancer. World J. Gastroenterol..

[5] Yang Q., Xu D., Yang Y., Lu S., Wang D., Wang L. (2024). Global, Regional, and National Burden of Gastric Cancer in Adolescents and Young Adults, 1990-2019: A Systematic Analysis for the Global Burden of Disease Study 2019. Am. J. Gastroenterol..

[6] Usui Y., Taniyama Y., Endo M., Koyanagi Y.N., Kasugai Y., Oze I., Ito H., Imoto I., Tanaka T., Tajika M. (2023). *Helicobacter pylori*, Homologous-Recombination Genes, and Gastric Cancer. N. Engl. J. Med..

[7] International Agency for Research on Cancer (IARC) (1994). Schistosomes, Liver Flukes and *Helicobacter pylori*. IARC Monogr. Eval. Carcinog. Risks Hum..

[8] Park J.Y., Georges D., Alberts C.J., Bray F., Clifford G., Baussano I. (2025). Global Lifetime Estimates of Expected and Preventable Gastric Cancers across 185 Countries. Nat. Med..

[9] Ito N., Tsujimoto H., Ueno H., Xie Q., Shinomiya N. (2020). *Helicobacter pylori*-Mediated Immunity and Signaling Transduction in Gastric Cancer. J. Clin. Med..

[10] Hatakeyama M. (2014). *Helicobacter pylori* CagA and Gastric Cancer: A Paradigm for Hit-and-Run Carcinogenesis. Cell Host Microbe.

[11] Yong X., Tang B., Li B.-S., Xie R., Hu C.-J., Luo G., Qin Y., Dong H., Yang S.-M. (2015). *Helicobacter pylori* Virulence Factor CagA Promotes Tumorigenesis of Gastric Cancer via Multiple Signaling Pathways. Cell Commun. Signal..

[12] Salvatori S., Marafini I., Laudisi F., Monteleone G., Stolfi C. (2023). *Helicobacter pylori* and Gastric Cancer: Pathogenetic Mechanisms. Int. J. Mol. Sci..

[13] Fang Z., Zhang W., Wang H., Zhang C., Li J., Chen W., Xu X., Wang L., Ma M., Zhang S. (2024). *Helicobacter pylori* Promotes Gastric Cancer Progression by Activating the TGF-β/Smad2/EMT Pathway Through HKDC1. Cell. Mol. Life Sci..

[14] Sozzi M., Valentini M., Figura N., De Paoli P., Tedeschi R.M., Gloghini A., Serraino D., Poletti M., Carbone A. (1998). Atrophic Gastritis and Intestinal Metaplasia in *Helicobacter pylori* Infection: The Role of CagA Status. Am. J. Gastroenterol..

[15] Winter J.A., Letley D.P., Cook K.W., Rhead J.L., Zaitoun A.A.M., Ingram R.J.M., Amilon K.R., Croxall N.J., Kaye P.V., Robinson K. (2014). A Role for the Vacuolating Cytotoxin, VacA, in Colonization and *Helicobacter pylori*-Induced Metaplasia in the Stomach. J. Infect. Dis..

[16] Abdullah M., Greenfield L.K., Bronte-Tinkew D., Capurro M.I., Rizzuti D., Jones N.L. (2019). VacA Promotes CagA Accumulation in Gastric Epithelial Cells during *Helicobacter pylori* Infection. Sci. Rep..

[17] He L., Zhang X., Zhang S., Wang Y., Hu W., Li J., Liu Y., Liao Y., Peng X., Li J. (2025). *H. pylori*-Facilitated TERT/Wnt/β-Catenin Triggers Spasmolytic Polypeptide-Expressing Metaplasia and Oxyntic Atrophy. Adv. Sci..

[18] Liu S., Zhang N., Ji X., Yang S., Zhao Z., Li P. (2025). *Helicobacter pylori* CagA Promotes Gastric Cancer Immune Escape by Upregulating SQLE. Cell Death Dis..

[19] Takahashi-Kanemitsu A., Knight C.T., Hatakeyama M. (2020). Molecular Anatomy and Pathogenic Actions of *Helicobacter pylori* CagA That Underpin Gastric Carcinogenesis. Cell. Mol. Immunol..

[20] Xiao S., Shen Y., Zhang M., Liu X., Cai T., Wang F. (2025). VacA Promotes Pyroptosis via TNFAIP3/TRAF1 Signaling to Induce Onset of Atrophic Gastritis. Microbiol. Res..

[21] Cao J., Yao M., Wang K., Qin L., Zhang Q., Zhang H., Wei J., Qu C., Xue C., Miao J. (2025). Sea Cucumber Fucoidan Inhibits *Helicobacter pylori* Gastritis via MAPK/NF-κB Signaling and Gut Microbiota Modulation. J. Agric. Food Chem..

[22] Zheng C., Du K., Tan B., Zhong G., Shao Z., Hu G., Wang Y., Meng D. (2026). Amelioration of *Helicobacter pylori*-Induced Gastric Injury by *Artemisia argyi* Folium Total Extract, Its Total Flavonoids, and Eupatilin Through Suppression of NF-κB Signaling and Virulence Factors. J. Ethnopharmacol..

[23] Chen Z.-W., Dong Z.-B., Xiang H.-T., Chen S.-S., Yu W.-M., Liang C. (2024). *Helicobacter pylori* CagA Protein Induces Gastric Cancer Stem Cell-like Properties Through the Akt/FOXO3a Axis. J. Cell. Biochem..

[24] Greaves M. (2010). Cancer Stem Cells: Back to Darwin?. Semin. Cancer Biol..

[25] Kubo S., Ninomiya R., Kajiwara T., Tokunaga A., Matsuda S., Murakami K., Yamaoka Y., Aigaki T., Hamada F. (2025). *Helicobacter pylori* Virulence Factor CagA Promotes Snail-Mediated Epithelial-Mesenchymal Transition and Invasive Behavior by Downregulating Semaphorin 5A in Gastric Epithelial Cells. Biochem. Biophys. Res. Commun..

[26] Robinson K., Atherton J.C. (2021). The Spectrum of *Helicobacter*-Mediated Diseases. Annu. Rev. Pathol..

[27] Álvarez-Aldana A., Beltrán-Angarita L., Guaca-González Y.M., Velandia-López M.A., Boyanova L. (2026). Levofloxacin and Rifampin Resistance in *Helicobacter pylori* Isolates from Central-Western Colombia: Role of gyrA Mutations in Fluoroquinolone Resistance. Antibiotics.

[28] Heo J., Jeon S.W. (2014). Optimal Treatment Strategy for *Helicobacter pylori*: Era of Antibiotic Resistance. World J. Gastroenterol..

[29] Han L., Wang R., Zhang X., Yu X., Zhou L., Song T., Deng X., Zhang Y., Zhang L., Bai C. (2019). Advances in Processing and Quality Control of Traditional Chinese Medicine *Coptidis rhizoma* (Huanglian): A Review. J. AOAC Int..

[30] Kong W.-J., Zhao Y.-L., Xiao X.-H., Wang J.-B., Li H.-B., Li Z.-L., Jin C., Liu Y. (2009). Spectrum-Effect Relationships Between Ultra Performance Liquid Chromatography Fingerprints and Anti-Bacterial Activities of *Rhizoma coptidis*. Anal. Chim. Acta.

[31] Wu J., Luo Y., Deng D., Su S., Li S., Xiang L., Hu Y., Wang P., Meng X. (2019). Coptisine from *Coptis chinensis* Exerts Diverse Beneficial Properties: A Concise Review. J. Cell. Mol. Med..

[32] Fan J., Zhang K., Jin Y., Li B., Gao S., Zhu J., Cui R. (2019). Pharmacological Effects of Berberine on Mood Disorders. J. Cell. Mol. Med..

[33] Ma B.-L., Ma Y.-M., Shi R., Wang T.-M., Zhang N., Wang C.-H., Yang Y. (2010). Identification of the Toxic Constituents in *Rhizoma coptidis*. J. Ethnopharmacol..

[34] Misra A., Chaudhary M.K., Rawat P., Tripathi D., Barik S.K., Srivastava S. (2024). Benzyl-Isoquinoline Alkaloids Rich Extract of Coptis Teeta Wall., Exhibit Potential Efficacy in Calcium-Oxalate and Uric-Acid Linked Metabolic Disorders. Fitoterapia.

[35] Zhao Q., Huang S., Yang L., Chen T., Qiu X., Huang R., Dong L., Liu W. (2024). Biomarkers and *Coptis chinensis* Activity for Rituximab-Resistant Diffuse Large B-Cell Lymphoma: Combination of Bioinformatics Analysis, Network Pharmacology and Molecular Docking. Technol. Health Care Off. J. Eur. Soc. Eng. Med..

[36] Zheng Y., Zhang M., Wu X., Tan R., Jiang H. (2024). *Coptis chinensis* Franch: Substance Basis, Mechanism of Action and Quality Control Standard Revealed Based on the Q-Marker Concept and New Strategy of Systemic Pharmacology and Biosynthesis Research. Curr. Top. Med. Chem..

[37] Zhu X., Wei Y., Yang B., Yin X., Guo X. (2020). The Mitohormetic Response as Part of the Cytoprotection Mechanism of Berberine: Berberine Induces Mitohormesis and Mechanisms. Mol. Med..

[38] Wang J., Wang L., Lou G.-H., Zeng H.-R., Hu J., Huang Q.-W., Peng W., Yang X.-B. (2019). *Coptidis rhizoma*: A Comprehensive Review of Its Traditional Uses, Botany, Phytochemistry, Pharmacology and Toxicology. Pharm. Biol..

[39] Wu X., Li X., Dang Z., Jia Y. (2018). Berberine Demonstrates Anti-Inflammatory Properties in *Helicobacter pylori*-Infected Mice with Chronic Gastritis by Attenuating the Th17 Response Triggered by the B Cell-Activating Factor. J. Cell. Biochem..

[40] Feng X., Sureda A., Jafari S., Memariani Z., Tewari D., Annunziata G., Barrea L., Hassan S.T.S., Šmejkal K., Malaník M. (2019). Berberine in Cardiovascular and Metabolic Diseases: From Mechanisms to Therapeutics. Theranostics.

[41] Tian E., Sharma G., Dai C. (2023). Neuroprotective Properties of Berberine: Molecular Mechanisms and Clinical Implications. Antioxidants.

[42] Liu Q., Tang J., Chen S., Hu S., Shen C., Xiang J., Chen N., Wang J., Ma X., Zhang Y. (2022). Berberine for Gastric Cancer Prevention and Treatment: Multi-Step Actions on the Correa’s Cascade Underlie Its Therapeutic Effects. Pharmacol. Res..

[43] Chen H.-B., Luo C.-D., Liang J.-L., Zhang Z.-B., Lin G.-S., Wu J.-Z., Li C.-L., Tan L.-H., Yang X.-B., Su Z.-R. (2017). Anti-Inflammatory Activity of Coptisine Free Base in Mice Through Inhibition of NF-κB and MAPK Signaling Pathways. Eur. J. Pharmacol..

[44] Lu Q., Tang Y., Luo S., Gong Q., Li C. (2023). Coptisine, the Characteristic Constituent from *Coptis chinensis*, Exhibits Significant Therapeutic Potential in Treating Cancers, Metabolic and Inflammatory Diseases. Am. J. Chin. Med..

[45] Liu L., Li J., He Y. (2020). Multifunctional Epiberberine Mediates Multi-Therapeutic Effects. Fitoterapia.

[46] Wu H., Xie X., Tang Q., Huang T., Tang X., Jiao B., Wang R., Zhu X., Ye X., Ma H. (2024). Epiberberine Inhibits *Helicobacter pylori* and Reduces Host Apoptosis and Inflammatory Damage by Down-Regulating Urease Expression. J. Ethnopharmacol..

[47] Tan L., Li C., Chen H., Mo Z., Zhou J., Liu Y., Ma Z., Xu Y., Yang X., Xie J. (2017). Epiberberine, a Natural Protoberberine Alkaloid, Inhibits Urease of *Helicobacter pylori* and Jack Bean: Susceptibility and Mechanism. Eur. J. Pharm. Sci. Off. J. Eur. Fed. Pharm. Sci..

[48] Li J., Chen X., Mao C., Xiong M., Ma Z., Zhu J., Li X., Chen W., Ma H., Ye X. (2025). Epiberberine Ameliorates MNNG-Induced Chronic Atrophic Gastritis by Acting on the EGFR-IL33 Axis. Int. Immunopharmacol..

[49] Li M., Yang J., Li J., Zhou Y., Li X., Ma Z., Li X., Ma H., Ye X. (2024). Epiberberine Induced P53/P21-Dependent G2/M Cell Cycle Arrest and Cell Apoptosis in Gastric Cancer Cells by Activating γ-Aminobutyric Acid Receptor-β3. Phytomedicine.

[50] Yu M., Ren L., Liang F., Zhang Y., Jiang L., Ma W., Li C., Li X., Ye X. (2020). Effect of Epiberberine from *Coptis chinensis* Franch on Inhibition of Tumor Growth in MKN-45 Xenograft Mice. Phytomed. Int. J. Phytother. Phytopharm..

[51] Yang S.B., Kim E.H., Kim S.H., Kim Y.H., Oh W., Lee J.-T., Jang Y.-A., Sabina Y., Ji B.C., Yeum J.H. (2018). Electrospinning Fabrication of Poly(Vinyl Alcohol)/*Coptis chinensis* Extract Nanofibers for Antimicrobial Exploits. Nanomaterials.

[52] Tarabasz D., Kukula-Koch W. (2020). Palmatine: A Review of Pharmacological Properties and Pharmacokinetics. Phytother. Res..

[53] Wang Y., Pei H., Chen W., Du R., Li J., He Z. (2023). Palmatine Protects PC12 Cells and Mice from Aβ25-35-Induced Oxidative Stress and Neuroinflammation via the Nrf2/HO-1 Pathway. Molecules.

[54] Ma W.-K., Li H., Dong C.-L., He X., Guo C.-R., Zhang C.-F., Yu C.-H., Wang C.-Z., Yuan C.-S. (2016). Palmatine from Mahonia Bealei Attenuates Gut Tumorigenesis in ApcMin/+ Mice via Inhibition of Inflammatory Cytokines. Mol. Med. Rep..

[55] Wang A., Guan B., Yu L., Liu Q., Hou Y., Li Z., Sun D., Xu H. (2024). Palmatine Protects Against Atherosclerosis by Gut Microbiota and Phenylalanine Metabolism. Pharmacol. Res..

[56] Rolle J., Asante D.O., Kok-Fong L.L., Boucetta H., Seidu T.A., Tai L.L.K., Alolga R.N. (2021). Jatrorrhizine: A Review of Its Pharmacological Effects. J. Pharm. Pharmacol..

[57] Zhang X., Wang G., Kuang W., Xu L., He Y., Zhou L., Zhang Y., Chen R., Li H., Fan T. (2024). Discovery and Evolution of Berberine Analogues as Anti-*Helicobacter pylori* Agents with Multi-Target Mechanisms. Bioorg. Chem..

[58] Zhou J.-T., Li C.-L., Tan L.-H., Xu Y.-F., Liu Y.-H., Mo Z.-Z., Dou Y.-X., Su R., Su Z.-R., Huang P. (2017). Inhibition of *Helicobacter pylori* and Its Associated Urease by Palmatine: Investigation on the Potential Mechanism. PLoS ONE.

[59] Tang Q., Ma Z., Tang X., Liu Y., Wu H., Peng Y., Jiao B., Wang R., Ye X., Ma H. (2023). Coptisine Inhibits *Helicobacter pylori* and Reduces the Expression of CagA to Alleviate Host Inflammation In Vitro and In Vivo. J. Ethnopharmacol..

[60] Imran M., Waqar S., Ogata K., Ahmed M., Noreen Z., Javed S., Bibi N., Bokhari H., Amjad A., Muddassar M. (2020). Identification of Novel Bacterial Urease Inhibitors Through Molecular Shape and Structure Based Virtual Screening Approaches. RSC Adv..

[61] Miller E.F., Maier R.J. (2014). Ammonium Metabolism Enzymes Aid *Helicobacter pylori* Acid Resistance. J. Bacteriol..

[62] Biagi F., Musiani F., Ciurli S. (2013). Structure of the UreD-UreF-UreG-UreE Complex in *Helicobacter pylori*: A Model Study. J. Biol. Inorg. Chem..

[63] Tsang K.L., Wong K.-B. (2022). Moving Nickel along the Hydrogenase-Urease Maturation Pathway. Met. Integr. Biometal Sci..

[64] Song Q., Wu H., Ma Z., Huang T., Zhu X., Zhang Z., Wu G., Manzoor R., Liu S., Wang Y. (2026). The Functional and Catalytic Landscape of Urease Reveals a Conserved Target Against *Helicobacter pylori*. Gut Microbes.

[65] Li C., Xie J., Chen X., Mo Z., Wu W., Liang Y., Su Z., Li Q., Li Y., Su Z. (2016). Comparison of *Helicobacter pylori* Urease Inhibition by *Rhizoma coptidis*, Cortex Phellodendri and Berberine: Mechanisms of Interaction with the Sulfhydryl Group. Planta Med..

[66] Li C., Huang P., Wong K., Xu Y., Tan L., Chen H., Lu Q., Luo C., Tam C., Zhu L. (2018). Coptisine-Induced Inhibition of *Helicobacter pylori*: Elucidation of Specific Mechanisms by Probing Urease Active Site and Its Maturation Process. J. Enzym. Inhib. Med. Chem..

[67] Fan T., Guo X., Zeng Q., Wei W., You X., Wang Y., Pang J., Song D. (2020). Synthesis and Structure-Activity Relationship of Palmatine Derivatives as a Novel Class of Antibacterial Agents Against *Helicobacter pylori*. Molecules.

[68] Villegas M., Ortega C., Gómez K., Cerda A., Sepúlveda R., Lara C., Bustamante L., Garcia D., Coppelli L., Hofmann E. (2025). High Prevalence of hefA Efflux Pump Overexpression in Isolates of *Helicobacter pylori* Resistant to Clarithromycin. Antibiotics.

[69] Huang Y.-Q., Huang G.-R., Wu M.-H., Tang H.-Y., Huang Z.-S., Zhou X.-H., Yu W.-Q., Su J.-W., Mo X.-Q., Chen B.-P. (2015). Inhibitory Effects of Emodin, Baicalin, Schizandrin and Berberine on hefA Gene: Treatment of *Helicobacter pylori*-Induced Multidrug Resistance. World J. Gastroenterol..

[70] Tang Q., Li X., Jiao B., Wu H., Li X., Ye X., Ma H. (2025). Evidences for the Mechanism of Anti-Inflammatory Effect of Coptisine Acting Against Clarithromycin-Resistant *Helicobacter pylori*. Phytomed. Int. J. Phytother. Phytopharm..

[71] Zhang D., Ke L., Ni Z., Chen Y., Zhang L.-H., Zhu S.-H., Li C.-J., Shang L., Liang J., Shi Y.-Q. (2017). Berberine Containing Quadruple Therapy for Initial *Helicobacter pylori* Eradication: An Open-Label Randomized Phase IV Trial. Medicine.

[72] Zhang J., Han C., Lu W.Q., Wang N., Wu S.R., Wang Y.X., Ma J.P., Wang J.H., Hao C., Yuan D.H. (2020). A Randomized, Multicenter and Noninferiority Study of Amoxicillin plus Berberine vs Tetracycline plus Furazolidone in Quadruple Therapy for *Helicobacter pylori* Rescue Treatment. J. Dig. Dis..

[73] Hu Q., Peng Z., Li L., Zou X., Xu L., Gong J., Yi P. (2019). The Efficacy of Berberine-Containing Quadruple Therapy on *Helicobacter pylori* Eradication in China: A Systematic Review and Meta-Analysis of Randomized Clinical Trials. Front. Pharmacol..

[74] Chen S., Shen W., Liu Y., Dong Q., Shi Y. (2023). Efficacy and Safety of Triple Therapy Containing Berberine, Amoxicillin, and Vonoprazan for *Helicobacter pylori* Initial Treatment: A Randomized Controlled Trial. Chin. Med. J..

[75] Sharaf M., Al-Laith Z.N., Khan T.U., Elkelish A., Abdel-Maksoud M.S., Elrefaei N.G., Alawam A.S., Liu C.-G. (2026). Mucoadhesive Lignin-Liposome Nanocarriers of Coptisine: A Multifunctional Strategy Against Antibiotic-Resistant *Helicobacter pylori* Biofilms. Bioorg. Chem..

[76] Johnson K.S., Ottemann K.M. (2018). Colonization, Localization, and Inflammation: The Roles of *H. pylori* Chemotaxis In Vivo. Curr. Opin. Microbiol..

[77] Maubach G., Vieth M., Boccellato F., Naumann M. (2022). *Helicobacter pylori*-Induced NF-κB: Trailblazer for Gastric Pathophysiology. Trends Mol. Med..

[78] Wang Y.-C. (2014). Medicinal Plant Activity on *Helicobacter pylori* Related Diseases. World J. Gastroenterol..

[79] Brandt S., Kwok T., Hartig R., König W., Backert S. (2005). NF-kappaB Activation and Potentiation of Proinflammatory Responses by the *Helicobacter pylori* CagA Protein. Proc. Natl. Acad. Sci. USA.

[80] Wu J., Zhang H., Hu B., Yang L., Wang P., Wang F., Meng X. (2016). Coptisine from *Coptis chinensis* Inhibits Production of Inflammatory Mediators in Lipopolysaccharide-Stimulated RAW 264.7 Murine Macrophage Cells. Eur. J. Pharmacol..

[81] Xie F., Yao S. (2025). Jatrorrhizine Attenuates Inflammatory Response in *Helicobacter pylori*-Induced Gastritis by Suppressing NLRP3 Inflammasomes and NF-κB Signaling Pathway. Arch. Microbiol..

[82] Osaki L.H., Bockerstett K.A., Wong C.F., Ford E.L., Madison B.B., DiPaolo R.J., Mills J.C. (2019). Interferon-γ Directly Induces Gastric Epithelial Cell Death and Is Required for Progression to Metaplasia. J. Pathol..

[83] Yang T., Wang R., Zhang J., Bao C., Zhang J., Li R., Chen X., Wu S., Wen J., Wei S. (2020). Mechanism of Berberine in Treating *Helicobacter pylori* Induced Chronic Atrophic Gastritis Through IRF8-IFN-γ Signaling Axis Suppressing. Life Sci..

[84] Sreesada P., Vandana, Krishnan B., Amrutha R., Chavan Y., Alfia H., Jyothis A., Venugopal P., Aradhya R., Suravajhala P. (2025). Matrix Metalloproteinases: Master Regulators of Tissue Morphogenesis. Gene.

[85] Chen X., Wang R., Bao C., Zhang J., Zhang J., Li R., Wu S., Wen J., Yang T., Wei S. (2020). Palmatine Ameliorates *Helicobacter pylori*-Induced Chronic Atrophic Gastritis by Inhibiting MMP-10 Through ADAM17/EGFR. Eur. J. Pharmacol..

[86] Cherdantseva L.A., Potapova O.V., Sharkova T.V., Belyaeva Y.Y., Shkurupiy V.A. (2014). Association of *Helicobacter pylori* and iNOS Production by Macrophages and Lymphocytes in the Gastric Mucosa in Chronic Gastritis. J. Immunol. Res..

[87] Pan L., Tang Q., Fu Q., Hu B., Xiang J., Qian J. (2005). Roles of Nitric Oxide in Protective Effect of Berberine in Ethanol-Induced Gastric Ulcer Mice. Acta Pharmacol. Sin..

[88] Yang H., Mou Y., Hu B. (2023). Discussion on the Common Controversies of *Helicobacter pylori* Infection. Helicobacter.

[89] Fei X., Li N., Xu X., Zhu Y. (2025). Macrophage Biology in the Pathogenesis of *Helicobacter pylori* Infection. Crit. Rev. Microbiol..

[90] Lu Y., Rong J., Lai Y., Tao L., Yuan X., Shu X. (2020). The Degree of *Helicobacter pylori* Infection Affects the State of Macrophage Polarization Through Crosstalk Between ROS and HIF-1α. Oxid. Med. Cell Longev..

[91] Yang T., Wang R., Liu H., Wang L., Li J., Wu S., Chen X., Yang X., Zhao Y. (2021). Berberine Regulates Macrophage Polarization Through IL-4-STAT6 Signaling Pathway in *Helicobacter pylori*-Induced Chronic Atrophic Gastritis. Life Sci..

[92] Lina T.T., Pinchuk I.V., House J., Yamaoka Y., Graham D.Y., Beswick E.J., Reyes V.E. (2013). CagA-Dependent Downregulation of B7-H2 Expression on Gastric Mucosa and Inhibition of Th17 Responses during *Helicobacter pylori* Infection. J. Immunol..

[93] Shah S.C., Piazuelo M.B., Kuipers E.J., Li D. (2021). AGA Clinical Practice Update on the Diagnosis and Management of Atrophic Gastritis: Expert Review. Gastroenterology.

[94] de Sousa Falcão H., Leite J.A., Barbosa-Filho J.M., de Athayde-Filho P.F., de Oliveira Chaves M.C., Moura M.D., Ferreira A.L., de Almeida A.B.A., Souza-Brito A.R.M., de Fátima Formiga Melo Diniz M. (2008). Gastric and Duodenal Antiulcer Activity of Alkaloids: A Review. Molecules.

[95] Hu C., Cao F., Jiang Y., Liu K., Li T., Gao Y., Li W., Han W. (2024). Molecular Insights into Chronic Atrophic Gastritis Treatment: *Coptis chinensis* Franch Studied via Network Pharmacology, Molecular Dynamics Simulation and Experimental Analysis. Comput. Biol. Med..

[96] Chen L., Wang X., Li J., Zhang L., Wu W., Wei S., Zou W., Zhao Y. (2024). Elucidation of the Mechanism of Berberine Against Gastric Mucosa Injury in a Rat Model with Chronic Atrophic Gastritis Based on a Combined Strategy of Multi-Omics and Molecular Biology. Front. Pharmacol..

[97] Chen X., Zhang J., Wang R., Liu H., Bao C., Wu S., Wen J., Yang T., Wei Y., Ren S. (2020). UPLC-Q-TOF/MS-Based Serum and Urine Metabonomics Study on the Ameliorative Effects of Palmatine on *Helicobacter pylori*-Induced Chronic Atrophic Gastritis. Front. Pharmacol..

[98] Jung J., Choi J.S., Jeong C.-S. (2014). Inhibitory Activities of Palmatine from *Coptis chinensis* Against *Helicobactor pylori* and Gastric Damage. Toxicol. Res..

[99] Sha Z.-G., Lin S., Fan Z.-L., Yang Z.-C., Gong Y.-T., Qin T., Yu R., He Y. (2025). Efficacy and Potential Mechanisms of Jatrorrhizine on MNNG-Induced Chronic Atrophic Gastritis in Rats Based on Serological Metabolomics and Molecular Docking. Sci. Rep..

[100] Wang L., Xie L. (2025). Berberine Alleviates 1-Methyl-3-Nitro-1-Nitrosoguanidine- Induced Chronic Atrophic Gastritis in Rats. Turk. J. Gastroenterol. Off. J. Turk. Soc. Gastroenterol..

[101] Jiang Q., Fan G., Wu K. (2025). Potential Action Mechanism of Erianin in Relieving MNNG-Triggered Chronic Atrophic Gastritis. Cell Biochem. Biophys..

[102] Tong Y., Zhao X., Wang R., Li R., Zou W., Zhao Y. (2021). Therapeutic Effect of Berberine on Chronic Atrophic Gastritis Based on Plasma and Urine Metabolisms. Eur. J. Pharmacol..

[103] Tong Y., Liu L., Wang R., Yang T., Wen J., Wei S., Jing M., Zou W., Zhao Y. (2021). Berberine Attenuates Chronic Atrophic Gastritis Induced by MNNG and Its Potential Mechanism. Front. Pharmacol..

[104] Liu K., Huang H., Xiong M., Wang Q., Chen X., Feng Y., Ma H., Chen W., Li X., Ye X. (2024). IL-33 Accelerates Chronic Atrophic Gastritis Through AMPK-ULK1 Axis Mediated Autolysosomal Degradation of GKN1. Int. J. Biol. Sci..

[105] Tran C.P., Scurr M., O’Connor L., Buzzelli J.N., Ng G.Z., Chin S.C.N., Stamp L.A., Minamoto T., Giraud A.S., Judd L.M. (2022). IL-33 Promotes Gastric Tumour Growth in Concert with Activation and Recruitment of Inflammatory Myeloid Cells. Oncotarget.

[106] Pisani L.F., Teani I., Vecchi M., Pastorelli L. (2023). Interleukin-33: Friend or Foe in Gastrointestinal Tract Cancers?. Cells.

[107] Zhou Y., Wang Q., Tang W., Ma Z., Yang Z., Li X., Chen W., Ma H., Ye X. (2024). Palmatine Ameliorates N-Methyl-N′-Nitrosoguanidine-Induced Chronic Atrophic Gastritis Through the STAT1/CXCL10 Axis. FASEB J..

[108] Ye X., Yang C., Xu H., He Q., Sheng L., Lin J., Wang X. (2024). Exploring the Therapeutic Mechanisms of *Coptidis rhizoma* in Gastric Precancerous Lesions: A Network Pharmacology Approach. Discov. Oncol..

[109] Yi T., Zhuang L., Song G., Zhang B., Li G., Hu T. (2015). Akt Signaling Is Associated with the Berberine-Induced Apoptosis of Human Gastric Cancer Cells. Nutr. Cancer.

[110] Nakonieczna S., Grabarska A., Gawel K., Wróblewska-Łuczka P., Czerwonka A., Stepulak A., Kukula-Koch W. (2022). Isoquinoline Alkaloids from *Coptis chinensis* Franch: Focus on Coptisine as a Potential Therapeutic Candidate Against Gastric Cancer Cells. Int. J. Mol. Sci..

[111] Li H.-L., Wu H., Zhang B.-B., Shi H.-L., Wu X.-J. (2016). MAPK Pathways Are Involved in the Inhibitory Effect of Berberine Hydrochloride on Gastric Cancer MGC 803 Cell Proliferation and IL-8 Secretion In Vitro and In Vivo. Mol. Med. Rep..

[112] Peng Z., Wangmu T., Li L., Han G., Huang D., Yi P. (2022). Combination of Berberine and Low Glucose Inhibits Gastric Cancer Through the PP2A/GSK3β/MCL-1 Signaling Pathway. Eur. J. Pharmacol..

[113] Li L.-L., Peng Z., Hu Q., Xu L.-J., Zou X., Huang D.-M., Yi P. (2022). Berberine Retarded the Growth of Gastric Cancer Xenograft Tumors by Targeting Hepatocyte Nuclear Factor 4α. World J. Gastrointest. Oncol..

[114] Xu M., Ren L., Fan J., Huang L., Zhou L., Li X., Ye X. (2022). Berberine Inhibits Gastric Cancer Development and Progression by Regulating the JAK2/STAT3 Pathway and Downregulating IL-6. Life Sci..

[115] Li L., Peng Z., Hu Q., Xu L., Zou X., Yu Y., Huang D., Yi P. (2020). Berberine Suppressed Tumor Growth Through Regulating Fatty Acid Metabolism and Triggering Cell Apoptosis via Targeting FABPs. Evid.-Based Complement. Altern. Med..

[116] Lin J.-P., Yang J.-S., Lee J.-H., Hsieh W.-T., Chung J.-G. (2006). Berberine Induces Cell Cycle Arrest and Apoptosis in Human Gastric Carcinoma SNU-5 Cell Line. World J. Gastroenterol..

[117] Yang Y., Zhang N., Li K., Chen J., Qiu L., Zhang J. (2018). Integration of microRNA-mRNA Profiles and Pathway Analysis of Plant Isoquinoline Alkaloid Berberine in SGC-7901 Gastric Cancers Cells. Drug Des. Dev. Ther..

[118] Wang J., Yang S., Cai X., Dong J., Chen Z., Wang R., Zhang S., Cao H., Lu D., Jin T. (2016). Berberine Inhibits EGFR Signaling and Enhances the Antitumor Effects of EGFR Inhibitors in Gastric Cancer. Oncotarget.

[119] Toshiyuki M., Reed J.C. (1995). Tumor Suppressor P53 Is a Direct Transcriptional Activator of the Human Bax Gene. Cell.

[120] Zhang X.-Z., Wang L., Liu D.-W., Tang G.-Y., Zhang H.-Y. (2014). Synergistic Inhibitory Effect of Berberine and D-Limonene on Human Gastric Carcinoma Cell Line MGC803. J. Med. Food.

[121] Yu S., Zhuo J., Hong X., Rao S., Ye Y., Kang D., Peng H., Zhuo H. (2026). Tumor-Derived Exosomal TAGLN2 Promotes Metastasis by Inducing Vascular Permeability and Angiogenesis via the NRP1/SEMA4D/YAP Axis. Adv. Sci..

[122] Manfioletti G., Fedele M. (2022). Epithelial-Mesenchymal Transition (EMT) 2021. Int. J. Mol. Sci..

[123] Kim M.A., Lee H.S., Lee H.E., Jeon Y.K., Yang H.K., Kim W.H. (2008). EGFR in Gastric Carcinomas: Prognostic Significance of Protein Overexpression and High Gene Copy Number. Histopathology.

[124] Terashima M., Kitada K., Ochiai A., Ichikawa W., Kurahashi I., Sakuramoto S., Katai H., Sano T., Imamura H., Sasako M. (2012). Impact of Expression of Human Epidermal Growth Factor Receptors EGFR and ERBB2 on Survival in Stage II/III Gastric Cancer. Clin. Cancer Res..

[125] Bessède E., Staedel C., Acuña Amador L.A., Nguyen P.H., Chambonnier L., Hatakeyama M., Belleannée G., Mégraud F., Varon C. (2014). *Helicobacter pylori* Generates Cells with Cancer Stem Cell Properties via Epithelial-Mesenchymal Transition-like Changes. Oncogene.

[126] Sougleri I.S., Papadakos K.S., Zadik M.P., Mavri-Vavagianni M., Mentis A.F., Sgouras D.N. (2016). *Helicobacter pylori* CagA Protein Induces Factors Involved in the Epithelial to Mesenchymal Transition (EMT) in Infected Gastric Epithelial Cells in an EPIYA- Phosphorylation-Dependent Manner. FEBS J..

[127] Lin S., Tsai S.-C., Lee C.-C., Wang B.-W., Liou J.-Y., Shyu K.-G. (2004). Berberine Inhibits HIF-1alpha Expression via Enhanced Proteolysis. Mol. Pharmacol..

[128] Lin J.-P., Yang J.-S., Wu C.-C., Lin S.-S., Hsieh W.-T., Lin M.-L., Yu F.-S., Yu C.-S., Chen G.-W., Chang Y.-H. (2008). Berberine Induced Down-Regulation of Matrix Metalloproteinase-1, -2 and -9 in Human Gastric Cancer Cells (SNU-5) In Vitro. In Vivo.

[129] Du H., Gu J., Peng Q., Wang X., Liu L., Shu X., He Q., Tan Y. (2021). Berberine Suppresses EMT in Liver and Gastric Carcinoma Cells Through Combination with TGFβR Regulating TGF-β/Smad Pathway. Oxid. Med. Cell Longev..

[130] Lu T.X., Rothenberg M.E. (2018). MicroRNA. J. Allergy Clin. Immunol..

[131] Hayashi Y., Tsujii M., Wang J., Kondo J., Akasaka T., Jin Y., Li W., Nakamura T., Nishida T., Iijima H. (2013). CagA Mediates Epigenetic Regulation to Attenuate Let-7 Expression in *Helicobacter pylori*-Related Carcinogenesis. Gut.

[132] Liu Z., Xiao B., Tang B., Li B., Li N., Zhu E., Guo G., Gu J., Zhuang Y., Liu X. (2010). Up-Regulated microRNA-146a Negatively Modulate *Helicobacter pylori*-Induced Inflammatory Response in Human Gastric Epithelial Cells. Microbes Infect..

[133] Babaeenezhad E., Rashidipour M., Jangravi Z., Moradi Sarabi M., Shahriary A. (2024). Cytotoxic and Epigenetic Effects of Berberine-Loaded Chitosan/Pectin Nanoparticles on AGS Gastric Cancer Cells: Role of the miR-185-5p/KLF7 Axis, DNMTs, and Global DNA Methylation. Int. J. Biol. Macromol..

[134] Yao J., Zhang H., Liu C., Chen S., Qian R., Zhao K. (2020). miR-450b-3p Inhibited the Proliferation of Gastric Cancer via Regulating KLF7. Cancer Cell Int..

[135] Leodolter A., Alonso S., González B., Ebert M.P., Vieth M., Röcken C., Wex T., Peitz U., Malfertheiner P., Perucho M. (2015). Somatic DNA Hypomethylation in *H. pylori*-Associated High-Risk Gastritis and Gastric Cancer: Enhanced Somatic Hypomethylation Associates with Advanced Stage Cancer. Clin. Transl. Gastroenterol..

[136] You H.-Y., Xie X.-M., Zhang W.-J., Zhu H.-L., Jiang F.-Z. (2016). Berberine Modulates Cisplatin Sensitivity of Human Gastric Cancer Cells by Upregulation of miR-203. In Vitro Cell Dev. Biol. Anim..

[137] Zhou X., Xu G., Yin C., Jin W., Zhang G. (2014). Down-Regulation of miR-203 Induced by *Helicobacter pylori* Infection Promotes the Proliferation and Invasion of Gastric Cancer by Targeting CASK. Oncotarget.

[138] Kou Y., Tong B., Wu W., Liao X., Zhao M. (2020). Berberine Improves Chemo-Sensitivity to Cisplatin by Enhancing Cell Apoptosis and Repressing PI3K/AKT/mTOR Signaling Pathway in Gastric Cancer. Front. Pharmacol..

[139] Pandey A., Vishnoi K., Mahata S., Tripathi S.C., Misra S.P., Misra V., Mehrotra R., Dwivedi M., Bharti A.C. (2015). Berberine and Curcumin Target Survivin and STAT3 in Gastric Cancer Cells and Synergize Actions of Standard Chemotherapeutic 5-Fluorouracil. Nutr. Cancer.

[140] Chen H., Zhang F., Li R., Liu Y., Wang X., Zhang X., Xu C., Li Y., Guo Y., Yao Q. (2020). Berberine Regulates Fecal Metabolites to Ameliorate 5-Fluorouracil Induced Intestinal Mucositis Through Modulating Gut Microbiota. Biomed. Pharmacother..

[141] Gong S., Zhang Y., Du W., Zhang C., Wu N., Zhang X., Ren Z., Zhang Y., Zhang P., Zhan C. (2026). An Oral Berberine Nanocapsule Platform Orchestrates Microbiota for Potent Gastric Cancer Chemotherapy. J. Nanobiotechnol..

[142] Guo X., Zhao X., Lu X., Zhao L., Zeng Q., Chen F., Zhang Z., Xu M., Feng S., Fan T. (2024). A Deep Learning-Driven Discovery of Berberine Derivatives as Novel Antibacterial Against Multidrug-Resistant *Helicobacter pylori*. Signal Transduct. Target. Ther..

[143] Sung J.J.Y., Coker O.O., Chu E., Szeto C.H., Luk S.T.Y., Lau H.C.H., Yu J. (2020). Gastric Microbes Associated with Gastric Inflammation, Atrophy and Intestinal Metaplasia 1 Year after *Helicobacter pylori* Eradication. Gut.

[144] Mills J.C., Goldenring J.R. (2017). Metaplasia in the Stomach Arises from Gastric Chief Cells. Cell. Mol. Gastroenterol. Hepatol..

[145] Wu S.-R., Liu Y.-H., Shi Y.-Q. (2021). Is Intestinal Metaplasia the Point of No Return in the Progression of Gastric Carcinogenesis?. Chin. Med. J..

[146] Kinoshita H., Hayakawa Y., Koike K. (2017). Metaplasia in the Stomach-Precursor of Gastric Cancer?. Int. J. Mol. Sci..

[147] Burclaff J., Osaki L.H., Liu D., Goldenring J.R., Mills J.C. (2017). Targeted Apoptosis of Parietal Cells Is Insufficient to Induce Metaplasia in Stomach. Gastroenterology.

[148] Zhang T., Wang Z., Zhang B., Tang X. (2026). Refining the Correa Cascade: Gastric Stem Cell Plasticity, Niche Remodelling, and Parallel Pathways to Neoplasia. Pharmacol. Res..

[149] Yasuda T., Wang Y.A. (2024). Gastric Cancer Immunosuppressive Microenvironment Heterogeneity: Implications for Therapy Development. Trends Cancer.

[150] He Z., Chen D., Wu J., Sui C., Deng X., Zhang P., Chen Z., Liu D., Yu J., Shi J. (2021). Yes Associated Protein 1 Promotes Resistance to 5-Fluorouracil in Gastric Cancer by Regulating GLUT3-Dependent Glycometabolism Reprogramming of Tumor-Associated Macrophages. Arch. Biochem. Biophys..

[151] Li P., Zhang H., Chen T., Zhou Y., Yang J., Zhou J. (2024). Cancer-Associated Fibroblasts Promote Proliferation, Angiogenesis, Metastasis and Immunosuppression in Gastric Cancer. Matrix Biol..

[152] Tang S., Che X., Wang J., Li C., He X., Hou K., Zhang X., Guo J., Yang B., Li D. (2025). T-bet+CD8+ T Cells Govern Anti-PD-1 Responses in Microsatellite-Stable Gastric Cancers. Nat. Commun..

[153] You L., Wang Q., Zhang T., Xiao H., Lv M., Lv H., Deng L., Zhang X., Zhang Y. (2025). USP14-IMP2-CXCL2 Axis in Tumor-Associated Macrophages Facilitates Resistance to Anti-PD-1 Therapy in Gastric Cancer by Recruiting Myeloid-Derived Suppressor Cells. Oncogene.

[154] Liu T., Zhao X., Cai T., Li W., Zhang M. (2025). Metabolic Reprogramming in *Helicobacter pylori* Infection: From Mechanisms to Therapeutics. Front. Cell. Infect. Microbiol..

[155] Liu Y.-T., Hao H.-P., Xie H.-G., Lai L., Wang Q., Liu C.-X., Wang G.-J. (2010). Extensive Intestinal First-Pass Elimination and Predominant Hepatic Distribution of Berberine Explain Its Low Plasma Levels in Rats. Drug Metab. Dispos..

[156] Pan G., Wang G.-J., Liu X.-D., Fawcett J.P., Xie Y.-Y. (2002). The Involvement of P-Glycoprotein in Berberine Absorption. Pharmacol. Toxicol..

[157] Cui H.-M., Zhang Q.-Y., Wang J.-L., Chen J.-L., Zhang Y.-L., Tong X.-L. (2015). Poor Permeability and Absorption Affect the Activity of Four Alkaloids from Coptis. Mol. Med. Rep..

[158] Chen N., Yang X.-Y., Guo C.-E., Bi X.-N., Chen J.-H., Chen H.-Y., Li H.-P., Lin H.-Y., Zhang Y.-J. (2018). The Oral Bioavailability, Excretion and Cytochrome P450 Inhibition Properties of Epiberberine: An In Vivo and In Vitro Evaluation. Drug Des. Dev. Ther..

[159] Chang C.-H., Huang W.-Y., Lai C.-H., Hsu Y.-M., Yao Y.-H., Chen T.-Y., Wu J.-Y., Peng S.-F., Lin Y.-H. (2011). Development of Novel Nanoparticles Shelled with Heparin for Berberine Delivery to Treat *Helicobacter pylori*. Acta Biomater..

[160] Cheng X., Geng J., Wang L., Ma X., Su Y., Arif M., Liu C. (2023). Berberine-Loaded Mannosylerythritol Lipid-B Nanomicelles as Drug Delivery Carriers for the Treatment of *Helicobacter pylori* Biofilms In Vivo. Eur. J. Pharm. Biopharm..

[161] Shen Y., Zou Y., Chen X., Li P., Rao Y., Yang X., Sun Y., Hu H. (2020). Antibacterial Self-Assembled Nanodrugs Composed of Berberine Derivatives and Rhamnolipids Against *Helicobacter pylori*. J. Control. Release Off. J. Control. Release Soc..

[162] Li X.K., Motwani M., Tong W., Bornmann W., Schwartz G.K. (2000). Huanglian, a Chinese Herbal Extract, Inhibits Cell Growth by Suppressing the Expression of Cyclin B1 and Inhibiting CDC2 Kinase Activity in Human Cancer Cells. Mol. Pharmacol..

[163] Wang W., Gu W., He C., Zhang T., Shen Y., Pu Y. (2022). Bioactive Components of Banxia Xiexin Decoction for the Treatment of Gastrointestinal Diseases Based on Flavor-Oriented Analysis. J. Ethnopharmacol..

[164] Li X.-H., Xu J.-Y., Wang X., Liao L.-J., Huang L., Huang Y.-Q., Zhang Z.-F. (2023). BanXiaXieXin Decoction Treating Gastritis Mice with Drug-Resistant *Helicobacter pylori* and Its Mechanism. World J. Gastroenterol..

[165] Lin Y., Liu K., Chen J., Li T., Sun Z., Fan Y., Zou P., Liu Y., Wang C., Ma C. (2026). *Pinellia ternata*–*Coptidis rhizoma* Herb Pair Suppresses *Helicobacter pylori* Colonization via NLRP3-Mediated Pyroptosis. J. Ethnopharmacol..

[166] Yi Z., Jia Q., Wang Y., Zhang Y., Xie T., Ling J. (2023). Elian Granules Alleviate Precancerous Lesions of Gastric Cancer in Rats by Suppressing M2-Type Polarization of Tumor-Associated Macrophages Through NF-κB Signaling Pathway. BMC Complement. Med. Ther..

[167] Park H.-S., Wijerathne C.U.B., Jeong H.-Y., Seo C.-S., Ha H., Kwun H.-J. (2018). Gastroprotective Effects of Hwanglyeonhaedok-Tang Against *Helicobacter pylori*-Induced Gastric Cell Injury. J. Ethnopharmacol..

